# Mitochondrial regulation of acute extrafollicular B‐cell responses to COVID‐19 severity

**DOI:** 10.1002/ctm2.1025

**Published:** 2022-09-14

**Authors:** Tianyu Cao, Li Liu, Kelvin Kai‐Wang To, Chun‐Yu Lim, Runhong Zhou, Yue Ming, Ka‐Yi Kwan, Sulan Yu, Chun‐Yin Chan, Biao Zhou, Haode Huang, Yufei Mo, Zhenglong Du, Ruomei Gong, Luk‐Tsz Yat, Ivan Fan‐Ngai Hung, Anthony Raymond Tam, Wing‐Kin To, Wai‐Shing Leung, Thomas Shiu‐Hong Chik, Owen Tak‐Yin Tsang, Xiang Lin, You‐qiang Song, Kwok‐Yung Yuen, Zhiwei Chen

**Affiliations:** ^1^ AIDS Institute, Li Ka Shing Faculty of Medicine The University of Hong Kong Hong Kong Special Administrative Region People's Republic of China; ^2^ Department of Microbiology, Li Ka Shing Faculty of Medicine The University of Hong Kong Hong Kong Special Administrative Region People's Republic of China; ^3^ Department of Immunology Fourth Military Medical University Xi'an People's Republic of China; ^4^ State Key Laboratory of Emerging Infectious Diseases, Department of Microbiology The University of Hong Kong Hong Kong Special Administrative Region People's Republic of China; ^5^ School of Biomedical Sciences University of Hong Kong Hong Kong Special Administrative Region People's Republic of China; ^6^ Department of Medicine, Li Ka Shing Faculty of Medicine The University of Hong Kong Hong Kong Special Administrative Region People's Republic of China; ^7^ Department of Medicine Queen Mary Hospital Hong Kong Special Administrative Region People's Republic of China; ^8^ Department of Pathology Princess Margaret Hospital Hong Kong Special Administrative Region People's Republic of China; ^9^ Department of Medicine and Geriatrics Princess Margaret Hospital Hong Kong Special Administrative Region People's Republic of China; ^10^ Department of Dermatology Tangdu Hospital, Fourth Military Medical University Xi'an People's Republic of China; ^11^ School of Chinese Medicine The University of Hong Kong Hong Kong Special Administrative Region People's Republic of China; ^12^ Centre for Virology Vaccinology and Therapeutics Limited Hong Kong Special Administrative Region People's Republic of China

**Keywords:** B‐cell suppression, COVID‐19, mitochondrial dysfunction, SARS‐CoV‐2

## Abstract

**Background:**

Patients with COVID‐19 display a broad spectrum of manifestations from asymptomatic to life‐threatening disease with dysregulated immune responses. Mechanisms underlying the detrimental immune responses and disease severity remain elusive.

**Methods:**

We investigated a total of 137 APs infected with SARS‐CoV‐2. Patients were divided into mild and severe patient groups based on their requirement of oxygen supplementation. All blood samples from APs were collected within three weeks after symptom onset. Freshly isolated PBMCs were investigated for B cell subsets, their homing potential, activation state, mitochondrial functionality and proliferative response. Plasma samples were tested for cytokine concentration, and titer of Nabs, RBD‐, S1‐, SSA/Ro‐ and dsDNA‐specific IgG.

**Results:**

While critically ill patients displayed predominantly extrafollicular B cell activation with elevated inflammation, mild patients counteracted the disease through the timely induction of mitochondrial dysfunction in B cells within the first week post symptom onset. Rapidly increased mitochondrial dysfunction, which was caused by infection‐induced excessive intracellular calcium accumulation, suppressed excessive extrafollicular responses, leading to increased neutralizing potency index and decreased inflammatory cytokine production. Patients who received prior COVID‐19 vaccines before infection displayed significantly decreased extrafollicular B cell responses and mild disease.

**Conclusion:**

Our results reveal an immune mechanism that controls SARS‐CoV‐2‐induced detrimental B cell responses and COVID‐19 severity, which may have implications for viral pathogenesis, therapeutic interventions and vaccine development.

## Graphical abstract

- Severe COVID‐19 patients displayed rapid extrafollicular (aN and dnCS) B cell activation.
- Mild patients engaged mitochondrial dysfunction (MD) to suppress excessive extrafollicular responses.
- Intracellular calcium drives MD, leading to the suppression of B cell response.
- Low MD in B cells correlates with higher amounts of antibodies, inflammation and severe COVID‐19.

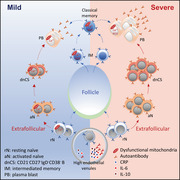

## 1 INTRODUCTION

COVID‐19 is characterized primarily by SARS‐CoV‐2 infection in airways and lungs with a broad spectrum of manifestations from asymptomatic to life‐threatening acute respiratory distress syndrome with aggressive proinflammatory response.^1^ A hallmark of SARS‐CoV‐2 infection is the dysregulated innate and adaptive immune response, including an early impairment of dendritic cell and CD8^+^ T‐cell function,^2^ and dysregulated monocytes/macrophages response.^3^, ^4^ SARS‐CoV‐2 infection elicits robust neutralizing antibody (NAb) titres with a rapid generation of B‐cell memory in most individuals.^5^, ^6^, ^7^ Humoral immune response, however, showed defective Bcl‐6(+) T follicular helper cell generation and the absence of germinal centres (GC) in some critically ill and deceased patients.^8^ Patients with severe COVID‐19 were marked by faster and higher viral‐specific serum antibody titre,^7^, ^9^ but lower NAbs potency index than mild patients.^10^ Notably, antibodies in severely ill patients often exhibited unique afucosylated Fc glycan, which enhanced its interactions with FcγIIIa and promoted the detrimental inflammatory response.^10^, ^11^, ^12^, ^13^ Moreover, defective B‐cell‐immune tolerance was reported in patients with life‐threatening COVID‐19 pneumonia. In these patients, the high prevalence of autoantibodies against cytokines, chemokines and complement components perturbed host‐immune response for viral control.^14^, ^15^, ^16^ These findings strongly suggested a dysregulated and pathogenic B‐cell responses in patients with poor disease outcomes.

Upon viral infection, activated B cells participate in either GC or extrafollicular (EF) response. GC responses are driven by T‐cell‐dependent (TD) antigen and T follicular helper cells in follicles. In follicles, somatically hypermutated B cells differentiate into memory B cells and antibody secreting cells. EF responses, in contrast, mostly are driven by T‐cell‐independent antigen and occur at EF sites, where B cells differentiate into plasmablasts (PBs).^17^ GC response often preceded by a short wave of EF proliferation, which is classified as the canonical response. Cytopathic viruses, such as influenza virus, tend to induce both EF and GC responses and produce NAbs more quickly than non‐cytopathic virus (e.g. LCMV).^18^, ^19^, ^20^ EF responses, however, were not always followed by GC reaction. Some pathogens, such as *Borrelia burgdorferi*, dominantly induce prolonged EF responses and suppress GCs reaction.^21^ High‐affinity NAbs are typically enriched in memory B cells and antibody secreting cells derived from GCs, contributing to long‐term protection from reinfection. Predominant and prolonged EF responses often result in a short‐term production of antibodies and memory B cells expressing BCRs with lower affinity against pathogen.^17^ How these B‐cell responses were regulated during acute SARS‐CoV‐2 infection remains incompletely understood.

The absence of GCs in patients with COVID‐19 suggests that SARS‐CoV‐2 infection probably elicits EF responses predominantly.^8^ In accordance with this study, some critically ill patients displayed robust EF B‐cell expansion, which correlated with large antibody secreting cells expansion and an early production of high amounts of SARS‐CoV‐2‐specific NAbs.^22^ Notably, the expansion of EF B cells was associated with significantly higher amounts of serum 9G4‐idiotype autoantibodies, which correlated with an elevated serum concentration of COVID‐19 disease biomarkers.^22^ These results indicated proinflammatory nature and a pathogenic role of EF B cells in COVID‐19, which is similar to EF‐driven responses previously described in systemic lupus erythematosus (SLE).^23^, ^24^ To date, immune mechanisms underlying the control of the abnormal EF B cell responses are largely unknown.

After showing that severely ill patients had accelerated and augmented antibody responses compared to mild patients during acute infection,^2^, ^7^ we sought to determine the mechanisms underlying SARS‐CoV‐2‐induced B‐cell dysregulation and COVID‐19 severity. By investigating 137 acutely infected patients, we provided evidence that abnormal EF response correlates with the breakdown of immune tolerance and disease severity. We also showed that mildly ill patients counteracted the disease process through a rapid induction of mitochondrial dysfunction (MD) in highly diverse B‐cell populations within the first week post‐symptom onset (PSO). The MD suppressed GC and EF B‐cell responses, lowered antibody production, increased IgG neutralizing potency index and predicted disease severity. Our results, therefore, demonstrated that mitochondrion plays a critical role in regulating acute EF B‐cell responses to reduce COVID‐19 severity.

## 2 RESULTS

### 2.1 Characteristics of patients with COVID‐19

We studied a total of 137 acute patients (AP) infected with SARS‐CoV‐2 who were recruited within 3‐week PSO. The first group included 64 patients between 8 July and 6 August 2020 for primary experiments, whereas the second group of 61 patients and third group of 12 patients were recruited between 23 July and 7 December 2020 and 28 January and 3 March 2022 for confirmation tests. All patients were admitted to the Hong Kong Queen‐Mary Hospital and Princess Margaret Hospital and were confirmed to be positive for SARS‐CoV‐2 by reverse‐transcription polymerase chain reaction (RT‐PCR).^25^ Among the first group of 64 APs, 15 were severe acute patients (S‐AP) who required oxygen supplementation, and the remaining 49 were mild acute patients (M‐AP). The median ages of S‐APs and M‐APs were 77‐ (interquartile range, 64–86) and 56 (interquartile range, 41–64)‐year old, respectively. Among these patients, hypertension and chronic comorbidities were less common in M‐APs (10/49, 20.4%; 24/49, 49%) than in S‐APs (9/15, 60%; 13/15, 86.7%). The rates of ICU admission and death in S‐APs (3/15, 20%; 5/15, 33.3%) were significantly higher than those in M‐APs (0/0, 0%; 0/0, 0%). The detailed clinical characteristics of these patients were shown in Table S1.

From the first 64 APs, a total of 73 blood samples were obtained, because 9 APs had two samples collected at different weeks PSO (Table S2). Of these 73 samples, 20 were obtained from S‐APs, whereas the remaining 53 were from M‐APs (Table S2). Laboratory data derived from plasma samples showed that serum levels of C‐reactive protein (CRP) and IL‐6, the two biomarkers of a poor prognosis for COVID‐19,^26^ were significantly higher in S‐APs than M‐APs (Figure S1a). S‐APs also showed significantly higher level of inflammation markers, including IL‐10, IFN‐α, TNF‐α and MCP‐1 at 1‐ or 2‐week PSO (Figure S1a). Viral load in nasal swabs, receptor‐binding domain (RBD)‐specific IgG and NAb titres were significantly lower in M‐APs (anti‐RBD titre: 490 [range, <50–5155] and NAb titre: 459.9 [range, <50–2994]) than in S‐APs (1228 [range, <50–12 886] and 1317 [range, 164–2766]) (Figure S1b,c), as previously described.^7^

### 2.2 Early and robust expansion of two CD21^−^CD27^−^ B cell subsets correlates with severe COVID‐19

**FIGURE 1:**
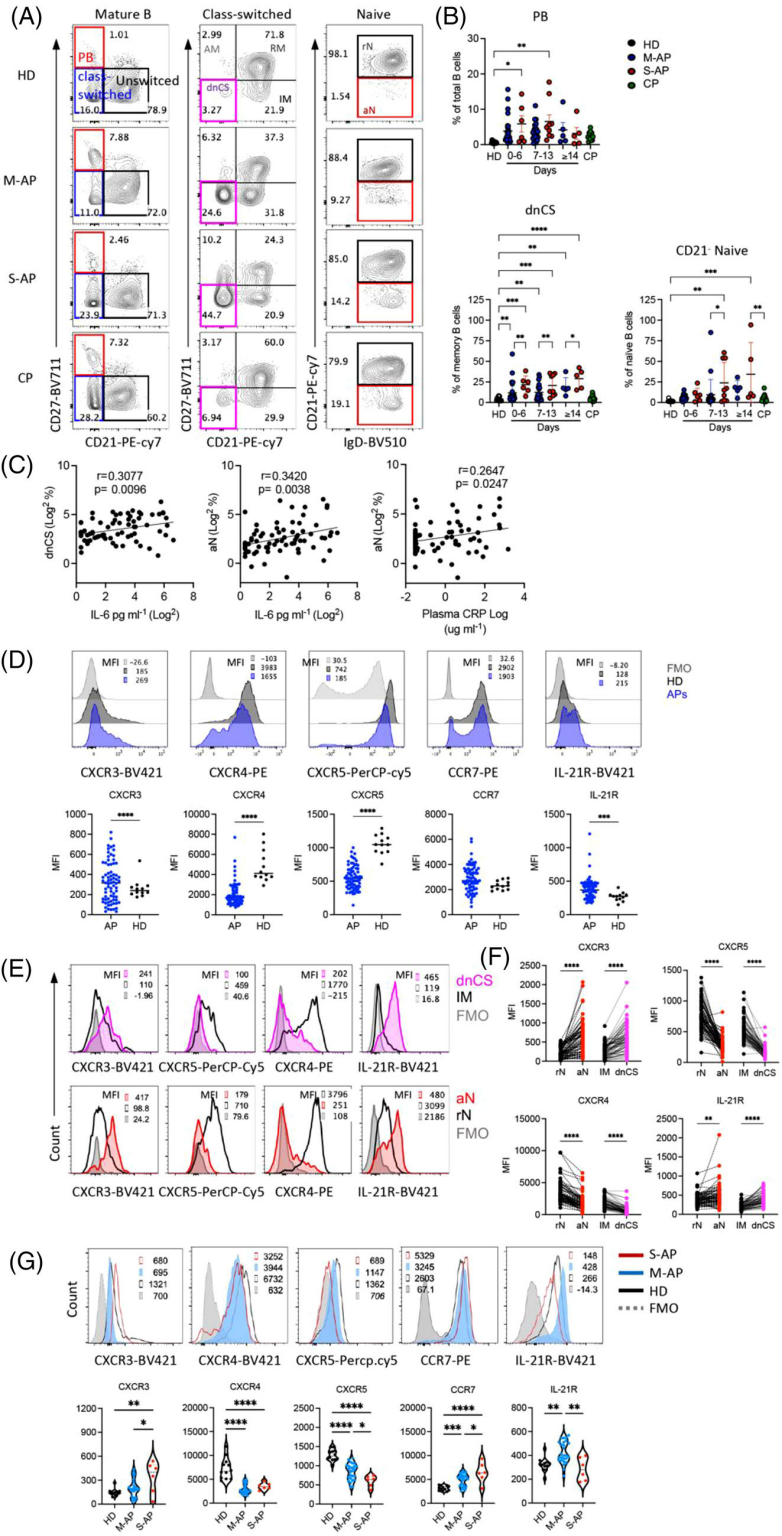
Patients with severe COVID‐19 exhibited more excessive extrafollicular (EF)‐like B‐cell activation than patients with mild COVID‐19 during acute infection. PBMCs from healthy donors (HDs) (*n* = 12), mild acute patients (M‐APs) (*n* = 50), severe acute patients (S‐APs) (*n* = 23) and convalescent patients (CPs) (*n* = 13) were analysed by flow cytometry. Representative patient samples were selected for display (A, D, E and F). (A) Primary population (plasmablasts [PB], naïve and class‐switched B cells) and secondary population (activated classical memory [AM], resting memory [RM], double negative class‐switched [dnCS], intermediated memory [IM], activated naïve [aN] and resting naïve [rN]) gating of representative patient samples. (B) Comparison of PB, dnCS and aN frequencies among HDs, CPs, M‐APs (days 0–6: *n* = 26; days 7–13: *n* = 22; days 14–21: *n* = 5) and S‐APs (days 0–6: *n* = 6; days 7–13: *n* = 8; days 14–21: *n* = 6) at weekly intervals post‐symptom onset (PSO). (C) Correlations of aN and dnCS cells with plasma IL‐6 or C‐reactive protein (CRP) concentrations. (D–F) Chemokine and cytokine receptor surface expression in total B cells (D), IM (black) versus dnCS (pink), and rN (black) versus aN (red) cells in APs (*n* = 73). FMO is shown in grey. (G) Chemokine and cytokine receptor surface expression on rN B cells in HDs (*n* = 12), M‐APs (*n* = 26) and S‐APs (*n* = 6) 1 week PSO. All samples were from the first group of APs. Data represent five measurements in (A and B), one measurement of each sample in (D–G). Statistical significance was determined using ordinary one‐way ANOVA and the two‐stage linear step‐up procedure of Benjamini, Krieger and Yekutieli, with a single pooled variance for (B and G), unpaired *t*‐test for (D), and paired *t*‐test for (F). *p ≤ .05, **p ≤ .01, ***p ≤ .001 and ****p ≤ .0001. *Source*: Correlation analyses were performed with a linear regression model using GraphPad Prism 8.0 software.

Using freshly isolated peripheral blood mononuclear cells (PBMCs) (*n* = 73) and the 12‐colour flow cytometry, we investigated B‐cell viability, subsets, their homing potential through integrin and chemokine receptor expression, activation state and mitochondrial functionality (Table S3). M‐APs and S‐APs were compared at weekly intervals PSO in 13 samples from convalescent patients (CPs) and 12 healthy donors (HDs) (Table S2). By gating on live mature B cells (Zombie^−^CD19^+^CD10^−^) for differential expression of IgD, CD38 and CD27, three primary B‐cell subsets were analysed, including CD38^++^IgD^−^ PBs, CD38^+/−^IgD^−^ Ig class‐switched B cells and IgD^+^CD38^+/−^ CD27^−^ naïve B cells^27^ (Figures 1A and S2a). We found that peripheral B cells in S‐APs exhibited a robust expansion of PBs compared with CPs and HDs (Figure 1A,B).

CD21 is constitutively expressed in peripheral resting naïve (rN) and CD27^+^ resting memory (RM) B cells. CD21 downregulation marks newly activated naïve (aN) and activated classical CD27^+^ memory (AM) cells in normal vaccination response or HIV and malaria infection.^28^, ^29^, ^30^ Loss of CD21 also identifies two blood B‐cell fractions, IgD^+^CD27^−^CD21^−^CD11c^+^ aN and IgD^−^CD27^−^CD21^−^CD11C^+^ DN2 cells. These two populations were derived from the EF pathway, responsible for increased autoantibodies in diseases, including SLE, Sjogren's syndrome and rheumatoid arthritis.^31^, ^32^, ^33^ Importantly, the expansion of aN and DN2 B cell populations was observed in patients with COVID‐19 and correlates with disease severity.^22^ We, therefore, fractioned class‐switched B cells for secondary subsets based on CD21 and CD27 expression, including CD21^+^CD27^+^ classical RM, CD21^−^CD27^+^ AM, CD21^+^CD27^−^ intermediated memory (IM) and CD21^−^CD27^−^ double negative class‐switched (dnCS) B cells. Naïve B cells were fractioned into CD21^−^ aN and CD21^+^ rN B cells (Figures S2a and 1A).^27^, ^32^, ^34^ IM and rN B cells were characterized as early activated memory cells and rN B cells and GC populations.^22^, ^23^, ^32^ We detected robust and prolonged expansion of aN and dnCS cells in APs. The expansion of dnCS cells was visible at the first week PSO and persisted for at least 3 weeks (Figure 1A,B). Notably, the magnitude and dynamic of expanded B‐cell subpopulations were distinct between M‐APs and S‐APs. S‐APs showed a more robust and prolonged expansion of aN and dnCS B cells (Figure 1A,B). Moreover, the frequencies of aN and dnCS B cells positively correlated with biomarkers of IL‐6 and CRP concentrations (Figure 1C), suggesting the association of aN and dnCS B cells expansion with COVID‐19 severity.

### 2.3 Patients with severe COVID‐19 displayed enhanced extrafollicular B‐cell activation

We next performed a signature analysis focusing on the two distinct subsets dnCS and aN using markers defining EF‐activated B cells in patients with COVID‐19 and SLE.^22^, ^32^ CPs were not included because they showed no significant alteration in blood B‐cell subset composition. We found that B cells in APs showed a decrease in GC homing and a corresponding increase in homing to tissue, as indicated by the lack of CXCR5 and a higher expression level of CXCR3 compared to HDs (Figure 1D). The most significant alteration was observed on dnCS and aN cells, which expressed a significantly lower level of CXCR5 and higher levels of CXCR3 than IM and rN, respectively, in both M‐APs and S‐APs (Figures 1E,F and S3a). Moreover, compared to rN and IM cells, aN and dnCS B cells in APs expressed a lower level of CXCR4, which is critical for GC reaction, and higher level IL‐21R (Figure 1E,F), which promotes B cells to differentiate into PBs through the EF pathway in autoimmune disease.^35^, ^36^, ^37^, ^38^ Critically, a further analysis of additional markers for EF‐derived cell population in APs (*n* = 6), including FcRL5 and CD11C,^34^ showed consistently that dnCS cells expressed significantly higher levels of FcRL5 and CD11C than IM and dnCS cells in HDs (*n* = 6) (Figure S3b). Our data, therefore, demonstrated that aN and dnCS cells represent EF‐derived populations in APs. Patients with severe COVID‐19 had enhanced EF B‐cell activation (Figure 1B–E).

Interestingly, rN, the precursor of aN and dnCS,^39^ also showed a significant and persistent decrease in CXCR5 and CXCR4 and an increase in chemokine receptors for T‐zone retention (CCR7) in APs (Figures 1G and S3c). S‐APs showed a more significant decrease in CXCR5 expression and enhanced upregulation of CXCR3 on rN during the first week PSO (Figures 1G and S3c), suggesting a more rapid skewing of rN B cells during the initiation stage of the B‐cell response to SARS‐CoV‐2 infection in S‐APs.

### 2.4 M‐APs might engage mitochondrial dysfunction to counteract excessive EF B‐cell activation

**FIGURE 2:**
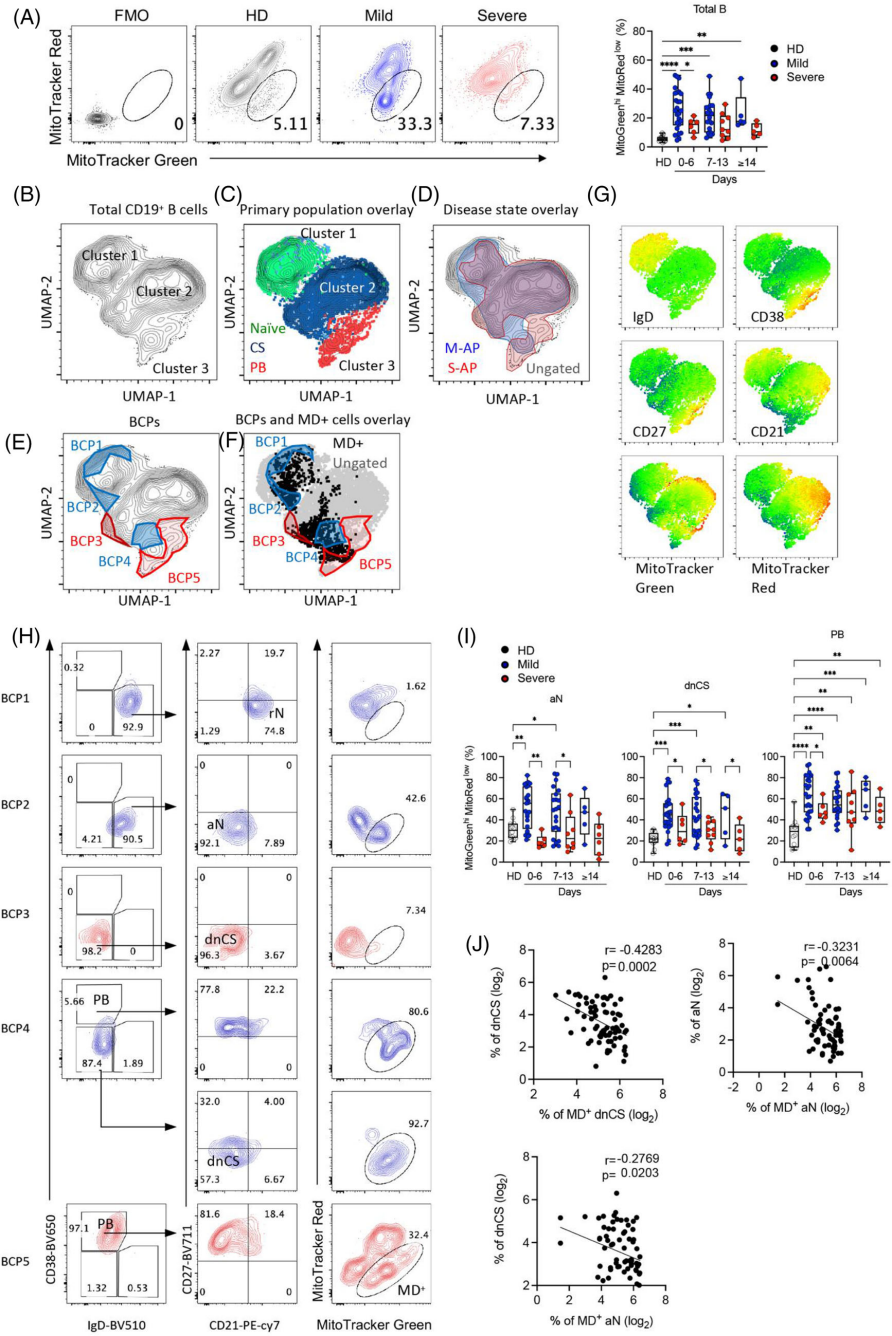
Increased mitochondrial dysfunction in activated naïve (aN) and double negative class‐switched (dnCS) cells in mild acute patients (M‐APs). (A) Comparison of the frequencies of MD^+^ B cells between healthy donors (HDs) (*n* = 12), M‐APs (*n* = 53) and severe acute patients (S‐APs) (*n* = 20) at weekly intervals post‐symptom onset (PSO). The left panel displays MD^+^ cell gating of representative patient samples. (B) Uniform manifold approximation and projection (UMAP) projection of composite patient samples. Composite sample was derived from 150 representative live B cells (Zobmie^−^CD19^+^) from patient PBMCs (*n* = 73) analysed with flow cytometry Panel 5 (Table S3). (C) Primary populations as gated in Figure 1A overlaid on the composite UMAP projection from (B). (D–F) Outlined regions represent the 90% equal probability contouring from the indicated classification. (D) Patient disease status overlaid on the composite UMAP projection from (B). (E) Regions of overlapping density were subtracted to display B‐cell populations (BCPs) indicating five unique populations. (F) Overlay of BCPs on the composite UMAP projection from (B) (grey) overlapping with B cells exhibiting mitochondrial dysfunction (MD) (black). (G) Heat maps of selected marker expression overlaid on the composite UMAP projection from (B). (H) Primary population (left panel, plasmablasts [PB], naïve and class‐switched memory B cells), secondary population (middle panel, resting naïve [rN], aN, dnCS) and MD^+^ cells gating of BCP1‐5. (I) Comparison of MD in indicated subpopulations among HDs (*n* = 12), M‐APs (days 0–6: *n* = 26; days 7–13: *n* = 22; days 14–21: *n* = 5) and S‐APs (days 0–6: *n* = 6; days 7–13: *n* = 8; days 14–21: *n* = 6) at weekly intervals PSO. (J) Correlation of the aN and dnCS cell frequency with MD magnitude in indicated populations in APs (*n* = 73). PBMCs are from the first group of APs and HDs in Figure 1 (Table S2). Statistical significance was determined using ordinary one‐way ANOVA and the two‐stage linear step‐up procedure of Benjamini, Krieger and Yekutieli, with a single pooled variance for (A and I). *p ≤ .05, **p ≤ .01, ***p ≤ .001 and ****p ≤ .0001. *Source*: Correlation analyses were performed with a linear regression model using GraphPad Prism 8.0 software.

The mitochondrial membrane potential is the driving force for ATP production essential for B‐cell activation and survival.^40^ Induction of MD often occurs in activated B cells and causes B‐cell apoptosis and death.^41^ We previously demonstrated that MD associates with T‐cell lymphocytopenia and impaired function during acute SARS‐CoV‐2 infection.^42^ We, therefore, sort to investigate whether MD contributed to the reduced EF‐activated B cells in M‐APs. By measuring MitoTracker Red as a marker of Δψm and MitoTracker Green as a marker of the mitochondrial injury to distinguish between respiring mitochondria and dysfunctional mitochondria,^43^, ^44^ we found in M‐APs (*n* = 53) but not in S‐APs (*n* = 20) a significant and consistent increase in B cells with MD (MitoTracker Green^high^, MitoTracker Red^low^) during the acute phase of infection (Figure 2A. The elevated MD^+^ B‐cell frequency was consolidated by an analysis of the MitoGreen^high^ TMRM^Low^ population (TMRM, another indicator for mitochondrial membrane potential^41^, ^45^) of valinomycin‐treated freshly isolated PBMCs from HDs (Figure S4). As shown in Figure S4a–c, the frequency of MitoTracker Green^high^ MitoTracker Red^Low^ B cells correlated positively and significantly with that of MitoTracker Green^high^ TMRM^Low^ B cells. Besides, the phenotype of MD^+^ B cells from Valinomycin‐treated cells of HDs (*n* = 3) was supported by relatively lower ATP production and maximal respiration by the Seahorse XF Cell Mito Stress Test (Figure S4d–f). Furthermore, B cells of 30 APs and 3 HDs isolated from the cryopreserved PBMC vials were tested for MD and pooled for Seahorse XF Cell Mito Stress Test, and the results showed a consistently higher frequency of MD^+^ B cells, lower ATP production, maximal respiration and spare respiration capacity in APs than HDs (Figure S4g–i).

Subsequently, composite samples were created by a representative downsampling of flow cytometry results obtained from HDs (*n* = 12), M‐APs (*n* = 53) and S‐APs (*n* = 20). The results were then recombined for analysis. A uniform manifold approximation and projection (UMAP) algorithm for dimensionality reduction was applied to the composite sample to generate contour plots (Figures 2B and S5a,b). Three cell clusters were observed in the projection, consisting of unswitched naïve B cells in cluster 1, class‐switched B cells in cluster 2 and PBs in cluster 3 (Figure 2B, C, G) . Overlaying a 90% equal probability contour from M‐APs and S‐APs revealed phenotypic B‐cell separation between these two groups (Figures 2D  and S5b). We then removed overlapping densities to reveal only densities occupied by S‐APs or M‐APs groups (Figure 2E). We found five B‐cell populations (BCPs 1–5) distinguishing M‐APs and S‐APs (Figure 2E). Critically, two BCPs in M‐APs (BCP2 and 4) highlighted areas of B‐cell populations with increased MD (Figure 2F, G) , consisting of predominantly aN in BCP2, dnCS B cells and PBs in BCP4 (Figure 2H). In contrast, two BCPs in S‐APs (BCP3 and 5) highlighted areas of dnCS (PCP3) and PBs (BCP5) with low levels of MD (Figure 2F, H). Further analysis revealed that B‐cell populations involving canonical GC pathway, including rN, IM, AM and RM, showed intermediate levels of MD in M‐APs (Figures S5c and S5). Robust increase of MD predominantly occurred in aN, dnCS B cells and PBs in M‐APs within the first week PSO and persisted over the course of the study (Figures 2I and S5c–e). In contrast, only AM showed a slight increase in MD in S‐APs (Figure S5c,d). The increase of MD in aN and dnCS cells correlated with a decrease in the numbers of aN and dnCS B cells (Figure 2J). Moreover, by gating on Zombie^−^ live B cells, we found that APs displayed decreased B‐cell viability in comparison with HDs. M‐APs showed more significant decrease in B‐cell viability than S‐APs at 1‐week PSO. Moreover, increasing MD correlated with decreasing cell viability (S5f). Taken together, these data suggested that M‐APs but not S‐APs likely counteracted the excessive EF B‐cell expansion by rapidly inducing MD in these B‐cell populations during acute SARS‐CoV‐2 infection.

### 2.5 Mitochondrial dysfunction caused an early suppression of EF and follicular B‐cell activation and proliferation

**FIGURE 3:**
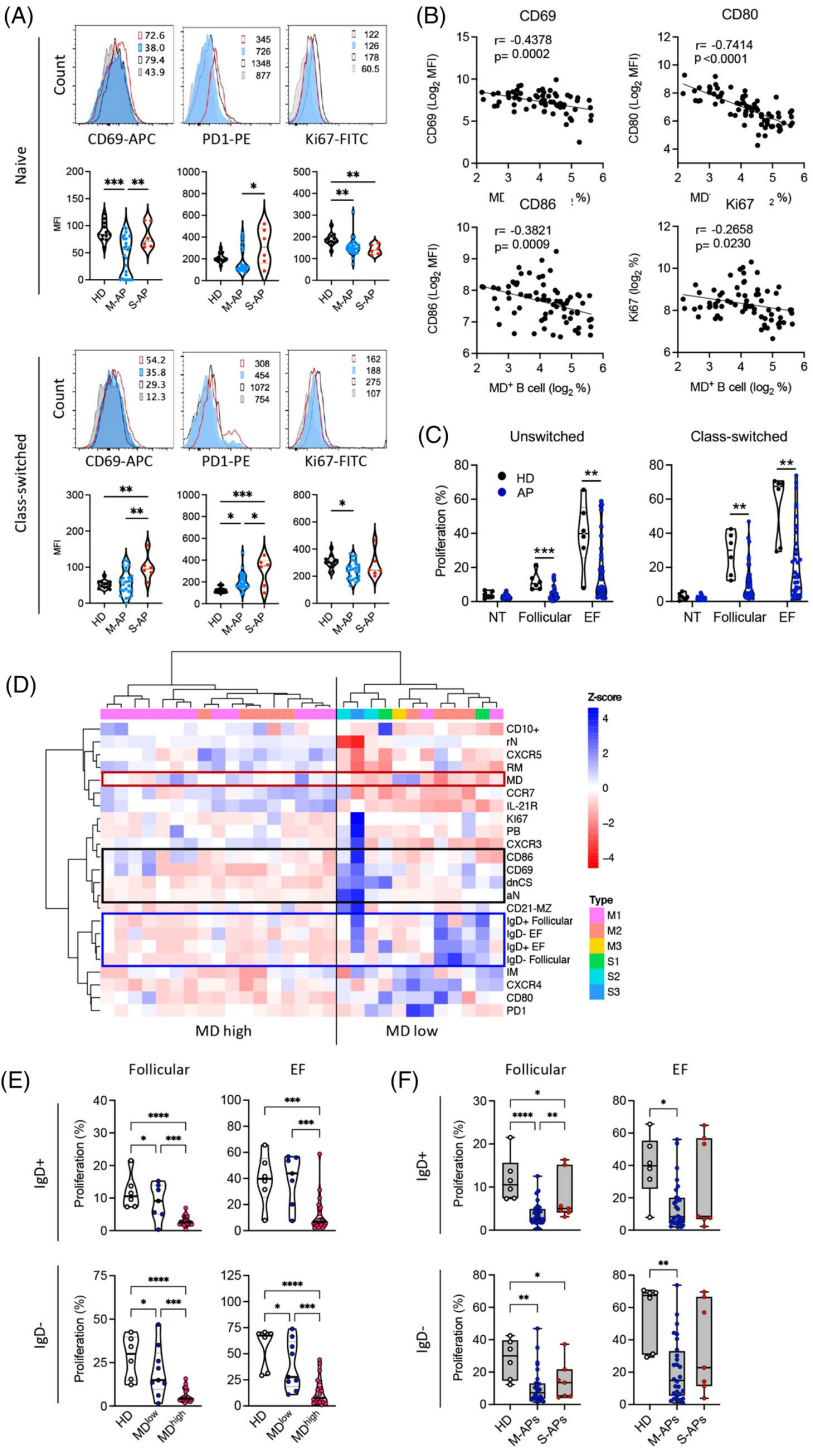
Mitochondrial dysfunction (MD) suppresses extrafollicular (EF) and follicular B‐cell activation and proliferation in mild acute patients (M‐APs). (A) Expression levels of CD69, PD‐1 and Ki67 on naïve and class‐switched memory B cells were compared between healthy donors (HDs) (*n* = 12), M‐APs (*n* = 26) and severe acute patients (S‐APs) (*n* = 6) 1 week post‐symptom onset (PSO). The upper panel displays representative patient samples. (B) Correlations of CD69, CD80, CD86 and Ki67 expression levels with the frequency of MD^+^ B cells in APs (*n* = 73). (C) Proliferative response of IgD^+^ and IgD^−^ B cells in APs (*n* = 35) and HDs (*n* = 6) to follicular (anti‐IgM/G plus IL‐2, IL‐10 and sCD40L) and EF (anti‐IgM/IgG plus CpG) stimulation. (D) Heat map of B‐cell phenotype and proliferative response in APs (*n* = 29). Dendrograms show the results of the multivariate clustering analysis of patients using Ward's method. The red box highlights MD. Black and blue boxes highlight EF populations and markers associated with B‐cell activation (black box) and the proliferative response (blue box). (E) Comparison of proliferative response of IgD^+^ B cells in HDs (*n* = 6), MD high APs (MD > 20%, *n* = 22) and MD low APs (MD < 20%, *n* = 7) and IgD^−^ B cells in HDs (*n* = 6); MD high APs (MD > 20%, *n* = 20) and MD low APs (MD < 20%, *n* = 9) to follicular and EF stimulation. (F) Comparison of proliferative response of IgD^+^ and IgD^−^ B cells in HDs (*n* = 6), M‐APs (days 0–6: *n* = 17; days 7–21: *n* = 11) and S‐APs (days 0–6: *n* = 2; days 7–21: *n* = 5). Flow cytometry data (A, B, D) were from the first group of APs and HDs in Figures 1, 3. Data of proliferative response were from 29 APs in the first group APs, plus 6 APs of the second group that had not flow cytometry analysis. Parts (D and E) were generated with 29 samples in (C). Samples were selected basing on their availability (Table S4). Statistical significance was determined using ordinary one‐way ANOVA and the two‐stage linear step‐up procedure of Benjamini, Krieger and Yekutieli, with a single pooled variance for (A, E and F) and using unpaired Student's *t*‐test for (C). *p ≤ .05, **p ≤ .01, ***p ≤ .001 and ****p ≤ .0001. *Source*: Correlation analyses were performed with a linear regression model using GraphPad Prism 8.0 software.

By measuring an expression of CD69, CD80, CD86, PD‐1 and Ki67, signatures distinguishing B‐cell activation and proliferation in M‐APs and S‐APs were identified during the first week PSO (Figure 3A). Naïve and class‐switched B cells expressed lower levels of CD69 and PD‐1 in M‐APs than in S‐APs (Figure 3A). Moreover, the expression of the proliferation marker Ki67 decreased in both naïve and class‐switched B cells in M‐APs but only in naïve B cells in S‐APs compared with HDs (Figure 3A). Furthermore, increased MD strongly correlated with a decreased expression of B‐cell activation and proliferation markers CD69, CD80, CD86 and Ki67 (Figure 3B). These results were in‐line with the causative role of MD in B‐cell apoptosis and death, and suppressed B‐cell response during acute SARS‐CoV‐2 infection.

We further assessed the proliferative capacity of B cells using IgD^+^ B cells (containing >95% naïve B cells) and IgD^−^ class‐switched B cells purified from freshly isolated PBMCs from APs (*n* = 35) and HDs (*n* = 6) (Figure S6a). Cells were cultured with stimulators that preferentially induce TD and GC (anti‐IgM/G/A plus IL‐2, IL‐10, and sCD40L) response or stimulators (anti‐IgM/IgG/A plus CpG) that preferentially promote EF response^46^, ^47^, ^48^ We found that all B‐cell samples from HDs showed proliferative responses with relatively consistent proliferation rates. In contrast, B‐cell samples from many APs showed a significantly suppressed proliferative response to both EF and GC stimuli (Figures 3C and S6b).

A hierarchical clustering analysis was then conducted using available samples for both B‐cell phenotype and proliferation capacity (*n* = 29) and revealed a clear separation of two groups, driven primarily by cells with high MD (Figure 3D). Reduced CD69 and CD80 expression and B‐cell proliferative response were consistently found in B cells with high MD. In contrast, the EF pathway activation, particularly an increased frequency of dnCS and aN, was observed in B cells with low MD (Figure 3D). Critically, B cells derived from patients with high frequencies of MD^+^ B cells exhibited a significantly lower proliferative response compared with the low MD group (Figure 3E) and increased MD significantly correlated with a decreasing proliferative response (Figure S6c). Concordantly, B cells derived from M‐APs showed a significantly suppressed proliferative response to both GC and EF stimuli (Figures 3F and S6b). The most significant suppression of proliferation was observed in the first week PSO (Figure S6d). In S‐APs, a decreased response was only observed to GC but not EF activation (Figure 3F). Taken together, these data demonstrated that SARS‐CoV‐2‐induced MD suppressed GC and EF responses in M‐APs, and lack of MD in EF populations accounts for the robust and persistent EF response in S‐APs.

### 2.6 Increased intracellular calcium levels caused mitochondrial dysfunction in patients with COVID‐19

**FIGURE 4:**
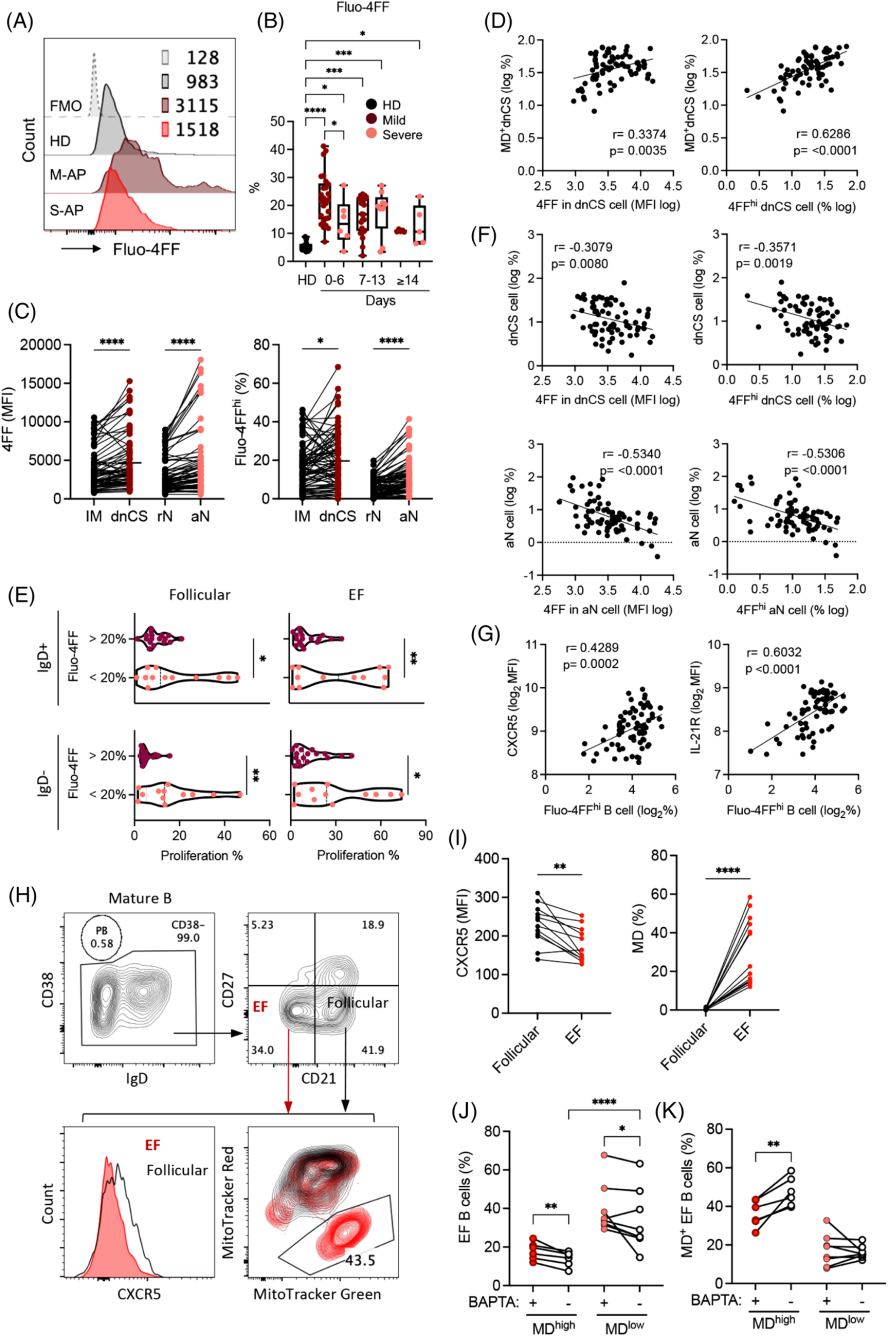
An increase in intracellular calcium levels causes mitochondrial dysfunction (MD) in patients with COVID‐19. (A and B) Intracellular calcium level measured using Fluo‐4FF staining and flow cytometry analysis. (A) Representative patient samples. (B) Frequency of Fluo‐4FF^hi^ B cells in healthy donors (HDs) (*n* = 12), mild acute patients (M‐APs) (days 0–6: *n* = 26; days 7–13: *n* = 22; days 14–21: *n* = 5) and severe acute patients (S‐APs) (days 0–6: *n* = 6; days 7–13: *n* = 8; days 14–21: *n* = 6) at weekly intervals post‐symptom onset (PSO). (C) Fluo‐4FF level (MFI) and Fluo‐4FF^hi^ B cells in intermediated memory (IM) (black) versus double negative class‐switched (dnCS) (purple), and resting naïve (rN) (black) versus activated naïve (aN) (red) cells in APs (*n* = 73). (D) Correlation of MD^+^ dnCS with the Fluo‐4FF level (MFI) and the frequency of Fluo‐4FF^hi^ dnCS B cells in APs (*n* = 73). (E) Proliferative response of IgD^+^ and IgD^−^ B cells to follicular and extrafollicular (EF) stimulation in Fluo‐4FF high (>20%, *n* = 17) and Fluo‐4FF low (<20%, *n* = 12) B cells in APs (*n* = 29). (F) Correlation of aN and dnCS frequencies with the Fluo‐4FF staining intensity and frequency of Fluo‐4FF^hi^ aN and dnCS B cells in APs (*n* = 73). (G) Correlation of CXCR5 and IL‐21R expression with the frequency of Fluo‐4FF^hi^ B cells in APS (*n* = 73). (H) CXCR5 expression and frequency of MD^+^ EF cells in representative samples. CD38^−/low^ B cells were gated based on CD21 and CD27 expression to obtain CD21^−^CD27^−^ EF populations containing both dnCS and aN cells, and CD21^+^CD27^−^ follicular populations, including rN and IM. (I) CXCR5 expression and frequency of MD^+^ cells in EF populations versus follicular populations in APs without BAPTA treatment (*n* = 14). (J and K) APs samples were divided into MD high (*n* = 6, MD = 39.6%–58.5%) and MD low (*n* = 8, MD = 12.1%–22.6%) groups as shown in (I). Frequencies of EF cells (J) and MD^+^ EF cells (K) in PBMC samples with and without BAPTA treatment were compared. Samples in (A–G) were from APs and HDs in the first patient group (*n* = 73). Data of proliferative response in (D) were from Figure 2E. Samples in (H–K) were M‐AP samples on days 0–14 PSO (*n* = 14) from the second group of APs. Data represent one measurement of each patient sample. Statistical significance was determined using ordinary one‐way ANOVA and the two‐stage linear step‐up procedure of Benjamini, Krieger and Yekutieli, with a single pooled variance for (B), and two‐tailed unpaired *t*‐test for (E), paired *t*‐test for (C, I, J and K). *p ≤ .05, **p ≤ .01, ***p ≤ .001 and ****p ≤ .0001

A well‐characterized cause of MD in B cells is the increased level of intracellular calcium.^42^, ^50^, ^51^ We, therefore, analysed fresh B cells in 73 PBMC samples derived from 64 APs by staining with the low affinity calcium indicator Fluo‐4FF. We found significantly increased intracellular Ca^2+^ levels in a broad range of B‐cell subsets in M‐APs compared with S‐APs within the first week PSO (Figures 4A,B and S7a,b). Notably, the EF subsets (dnCS and aN) exhibited significantly higher intracellular Ca^2+^ levels in comparison with follicular populations IM and rN B cells (Figure 4C). The intracellular Ca^2+^ levels and the frequencies of Fluo‐4FF^hi^ B cells were significantly and positively correlated with the frequencies of MD^+^ dnCS cells (Figure 4D) and inversely correlated with the B‐cell proliferative capacity and the magnitude of dnCS and aN cells (Figures 4E,F and S7c). Moreover, increased intracellular Ca^2+^ levels correlated strongly with markers associated with GC response, including increased CXCR5 and IL‐21R expression on B cells (Figures 1F and 4F).^40^, ^52^ The results indicated a role of excessive intracellular calcium in causing MD and likely in suppressing the EF response in patients with COVID‐19.

Subsequently, freshly isolated PBMCs from 14 newly recruited M‐APs were pretreated with the cell‐permeant calcium chelator BAPTA for 1 h to decrease cellular calcium levels so that we could determine the role of intracellular calcium in the induction of MD. B cells were subsequently cultured for 24 h and analysed for MD and related biomarkers. Untreated B cells from the same patients were cultured in parallel as controls. During the analysis, zombie^−^CD19^+^ live B cells were gated for CD38 expression, and CD38^−^ B cells were subsequently gated based on CD21 and CD27 expression to obtain CD21^−^CD27^−^ populations containing both dnCS and aN cells (Figures S7d and 4G). We did not observe any significant decrease of live cells after BAPTA treatment (Figure S7e). In the untreated control samples, EF cells displayed consistently lower levels of CXCR5 than follicular cells (CD21^+^CD27^−^) (Figure 4H,I). Moreover, an enrichment of MD^+^ cells was observed in EF cells compared with GC cells (Figure 4H,I). As shown in Figure 4I, AP samples were divided into two clusters driven by the magnitude of MD in untreated control samples. High magnitude of MD associated with low frequency of EF populations (Figure 4J). Upon comparison of samples with and without BAPTA treatment within the MD^high^ (*n* = 6, MD = 39.6%–58.5%) and MD^low^ group (*n* = 8, MD = 12.1%–22.6%), we found that treatment with BAPTA significantly decreased the magnitude of MD^+^ cells and increased the frequency of EF population in MD^high^ group (Figure 4J,K). In the low MD group, the increase of EF cell frequency was observed (Figure 4J). Our findings, therefore, revealed a role of infection‐induced excessive intracellular calcium in inducing high frequency of MD in EF cells among COVID‐19 patients.

### 2.7 Increased mitochondrial dysfunction in EF B cells underlie higher anti‐S1 IgG neutralizing potency index

**FIGURE 5:**
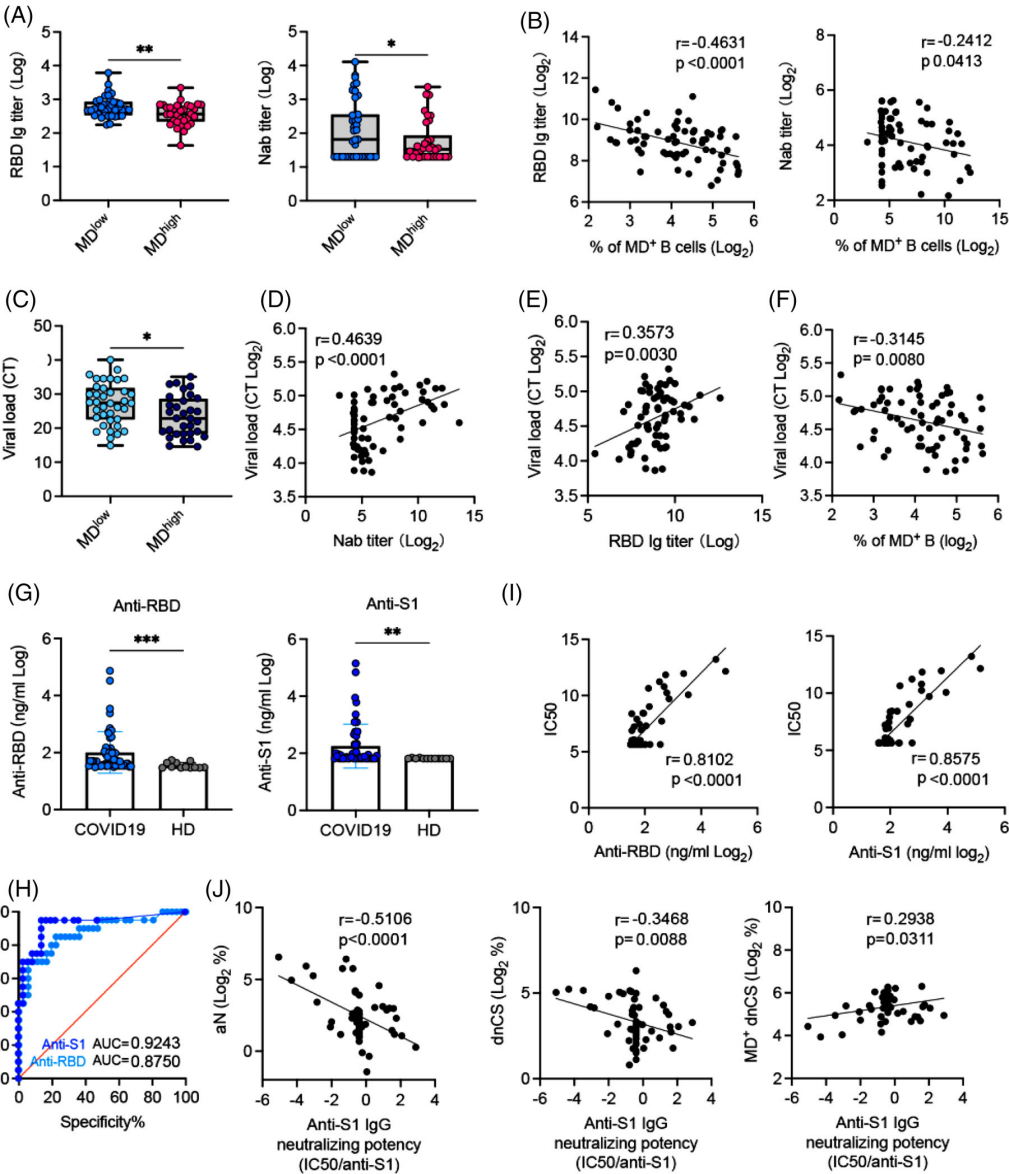
Higher mitochondrial dysfunction (MD) correlates with higher neutralizing potency index in patients with COVID‐19. (A) Receptor‐binding domain (RBD)‐specific IgG and neutralizing antibody (NAb) serum titres in MD high (MD > 20%, *n* = 34) and MD low (MD < 20% *n* = 39) patient sample groups. (B) Correlation of RBD‐specific IgG and NAb serum titres with the frequency of MD^+^ B cells. (C) Comparison of the viral load in nasal swabs between MD high (MD > 20%, *n* = 34) and MD low (MD < 20% *n* = 39) patient sample groups. (D–F) Correlations of the nasal swab viral load with NAb serum titres (D), RBD‐specific IgG (E) and MD magnitude of B cells in acute patients (APs) (*n* = 73) (F). (G) Anti‐RBD and anti‐S1 protein IgG serum concentration in APs (*n* = 53) and healthy donors (HDs) (*n* = 12). (H) Receiver operating curve (ROC) curve analysis of anti‐RBD and anti‐S1 IgG for the prediction of neutralization was performed; area under the curve (AUC) is indicated. (I) Correlation of anti‐RBD and anti‐S1 IgG with NAb serum titre in APs (*n* = 53). (J) Correlation of anti‐S1 IgG neutralizing potency index (IC_50_/anti‐S1 IgG) with the frequencies of activated naïve (aN), double negative class‐switched (dnCS) and MD^+^ dnCS B cells (*n* = 53). Samples in (A and C) were PBMCs and nasal swabs from the first patient group (*n* = 73). Samples in (G) were 53 plasma samples from the first group. Samples selection was based on their availability. Data of antibodies levels represent at least two measurements from each patient at each time point assessed. Statistical significance was determined using a two‐tailed unpaired *t*‐test for (A, C and G). ROC analysis was performed with the Mann–Whitney *U* test. *p ≤ .05, **p ≤ .01, and ***p ≤ .001. *Source*: Correlation analyses were performed with a linear regression model using GraphPad Prism 8.0 software.

**FIGURE 6:**
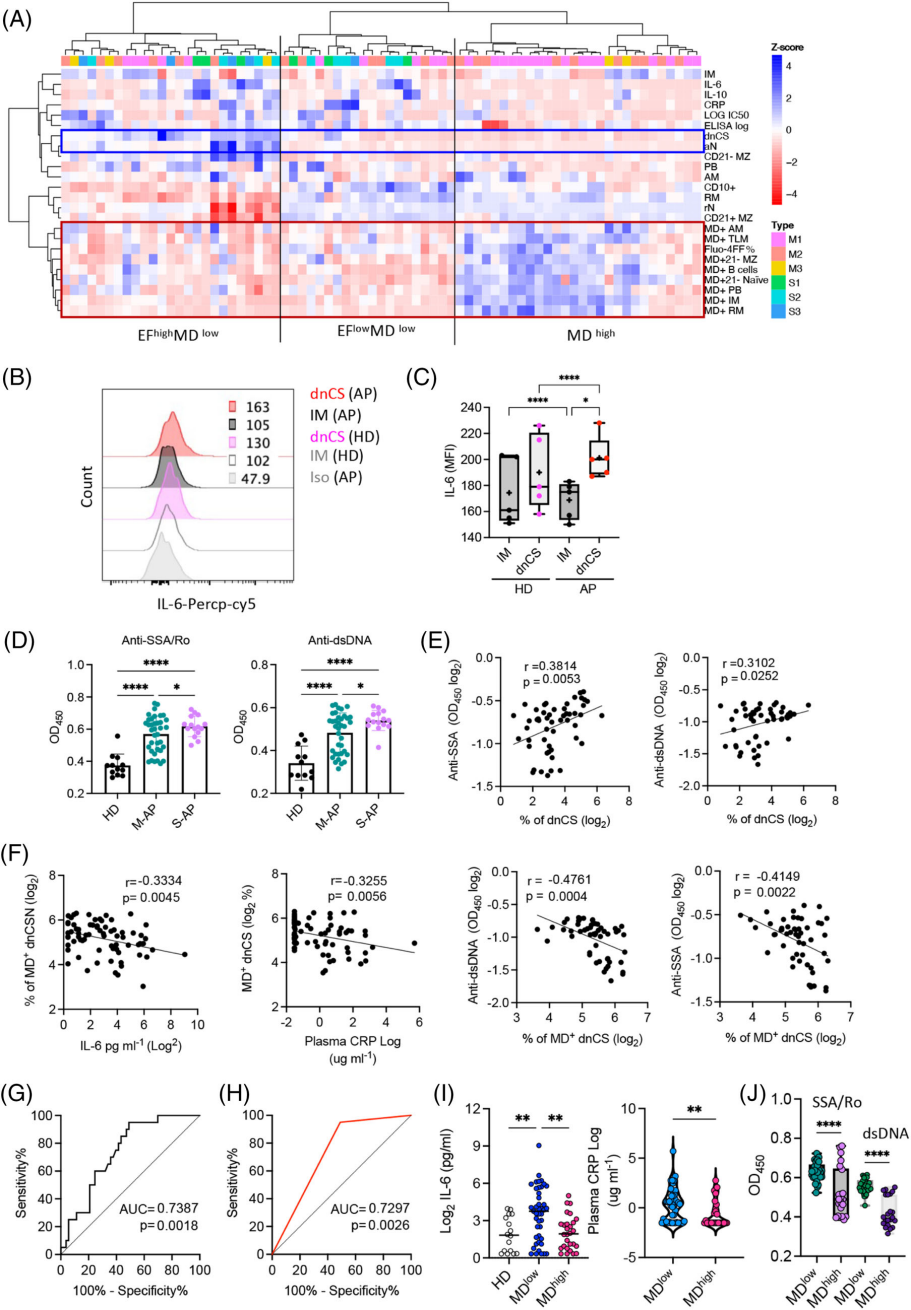
Lack of mitochondrial dysfunction correlates with severe COVID‐19. (A) Heat map of the B‐cell phenotype and serum antibody, cytokine and C‐reactive protein (CRP) concentrations in acute patients (APs) (*n* = 73). Dendrograms show the results of the multivariate clustering analysis of patients using Ward's method. The red box highlights MD^+^ populations and cellular calcium staining. Blue boxes highlight extrafollicular (EF) populations. (B and C) Expression levels of IL‐6 in double negative class‐switched (dnCS) and intermediated memory (IM) B cells were compared between healthy donors (HDs) (*n* = 5) and APs (*n* = 5). Part (B) displays representative patient samples. (D) Serum anti‐SSA/Ro and anti‐dsDNA autoantibody levels in plasma of HDs (*n* = 12), M‐APs (*n* = 37) and severe acute patients (S‐APs) (*n* = 16). (E) Correlation of serum autoantibodies levels with a frequency of dnCS B cells in APs (*n* = 53). Samples were selected basing on their availabilities. (F) Correlations of MD^+^ dnCS with plasma IL‐6 and CRP levels (*n* = 73) and anti‐SSA/Ro and anti‐dsDNA IgG1 levels (*n* = 53). (G) A receiver operating curve (ROC) analysis of MD^+^ B cells for the prediction of COVID‐19 severity (*n* = 73). Area under the curve (AUC) is indicated. The Mann–Whitney *U* test was used, and the two‐tailed *p* value is shown. (H) An ROC analysis showed an AUC of .729, with an MD^+^ B cell cut‐off 23% achieving a sensitivity of 95% and specificity of 50.94%. (I) Plasma IL‐6 and CRP concentrations in the HD (*n* = 16), mitochondrial dysfunction (MD) high (>23%, *n* = 45) and MD low (<23%, *n* = 28) APs. (J) Serum anti‐SSA/Ro and anti‐dsDNA antibody levels in MD high (MD > 23%, *n* = 30) and MD low (MD < 23% *n* = 23) patient sample groups. (B) Correlation of serum anti‐SSA/Ro and anti‐dsDNA antibody levels with the frequency of MD^+^ B‐cell populations in APs (*n* = 53). Data represent at least a single measurements from each patient at each time point assessed. Statistical significance was determined using ordinary one‐way ANOVA and the two‐stage linear step‐up procedure of Benjamini, Krieger and Yekutieli, with a single pooled variance for a and the left panels in (H) and a two‐tailed unpaired *t*‐test for (A and I). *p ≤ .05, **p ≤ .01 and ****p ≤ .0001. *Source*: Correlation analyses were performed using GraphPad Prism 8.0 software.

As high MD suppressed B‐cell proliferative responses (Figure 2), we sought to determine whether increased MD was associated with reduced levels of SARS‐CoV‐2‐specific NAbs in the 73 samples collected from our patients. By dividing APs samples into high MD (*n* = 34, MD > 20%) and low MD (*n* = 39, MD < 20%) groups, we found that serum RBD‐specific IgG and NAb titres were significantly lower in the high MD group than in the low MD group (Figure 5A). Accordingly, increased MD in B cells correlated with decreased RBD‐specific IgG and NAb titres (Figure 5B). Concordant with the decreased NAb activity, higher nasal swabs viral loads were found in M‐APs with high MD (Figure 5C). Moreover, higher viral loads correlated with lower serum NAb and RBD‐Ig titres and higher MD in B cells from APs (Figure 6D–F), thus confirming the suppressive effect of MD on NAb responses in patients with COVID‐19.

EF B‐cell populations often produce pathogen‐specific antibodies with lower affinity than GC‐derived antibodies.^17^ We, therefore, proceeded to analyse whether the increase of MD in EF B cells underlie higher IgG neutralizing potency index, which was recently described in mildly ill patients.^10^ We quantified a serum concentration of IgG against SARS‐CoV‐2 Spike protein S1 subunit and RBD in all available patients’ serum (*n* = 53) and found significant increases in IgG against RBD and S1 subunit of S protein in APs (*n* = 53) compared with HDs (*n* = 13) (Figure 5G). Upon analysing NAbs seropositivity and IgG antibody concentration using receiver operating curve (ROC), we found that anti‐S1 IgG was a better predictor of neutralization than anti‐RBD IgG in our patients (Figure 5H). Indeed, anti‐S1 IgG levels correlated nicely with neutralization (Figure 5I). We then calculated the neutralizing potency index (IC_50_/S1 IgG) for each serum using the similar approach previously described.^10^ The results showed that the neutralizing potency index was negatively and significantly correlated with the magnitude of aN and dnCS populations. Notably, the increased frequency of MD^+^ dnCS cells correlated with the neutralizing potency index positively (Figure 5J). Together, these findings demonstrated that increase of MD in dnCS B cells promoted the serum neutralizing potency index in our patients probably by reducing EF response and the proportion of antibodies with low affinity against S1.

### 2.8 Lack of mitochondrial dysfunction in EF B cells correlated closely with poorer disease outcomes

EF B‐cell activation was previously described as an the major contributors to inflammatory response^27^, ^53^, ^54^ and associated with autoantibodies production, IL‐6, CRP and autoantibody serum concentrations in patients with COVID‐19 (Figure S2c).^23^, ^24^, ^33^ We, therefore, sought to investigate whether increased MD was associated with reduced levels of inflammation, autoantibodies and disease severity. The hierarchical clustering analysis conducted using all PBMC samples tested revealed the separation of M‐APs in the area of APs with a high frequency of MD^+^ cells, low concentrations of IL‐6 and CRP, and a low frequency of EF populations. In contrast, S‐APs were distributed in MD low and IL‐6/CRP high clusters (Figure 6A). In agreement with this observation, using flow cytometry, we found that dnCS B cells expressed a significantly higher level of IL‐6 in APs (*n* = 5) (Figure 6B,C), and IL‐6 and CRP concentrations correlated with the frequencies of EF populations (Figure S2c). We next tested available APs serum samples and found significant higher serum concentrations of anti‐SSA/Ro and anti‐dsDNA antibodies in S‐APs (*n* = 15) and M‐APs (*n* = 37) than HDs (*n* = 12) (Figure 6D). Moreover, increasing concentrations of serum anti‐SSA/Ro and anti‐dsDNA correlated with the magnitude of EF B cells (Figure 6E). Importantly, the magnitude of MD^+^ EF populations was significantly correlated with a lower expression of the biomarkers IL‐6, CRP, anti‐SSA/Ro and anti‐dsDNA antibodies (Figure 6F), suggesting the relevance of the MD magnitude in reduced disease severity.

We then conducted an ROC analysis to determine optimal MD^+^ B cells% cut‐offs that distinguished critically ill patients from mild patients with high sensitivity and specificity (Figure 6G). An MD^+^ B cells frequency threshold of 23% achieved a sensitivity of 95% and a specificity of 50.94% in identifying critically ill patients (Figure 6H). Concordantly, both biomarkers, IL‐6 and CRP, and anti‐SSA/Ro and anti‐dsDNA antibodies displayed increased expression in the low MD group (MD < 23%, *n* = 45) (Figure 6I,J). Critically, APs in the low MD groups had a significantly higher incidence of oxygen supplementation requirements (37.8% vs. 3.7%, *p* = .002) and death (13.5% vs. 0%, *p* = .068) (Table S4). Together, these results indicated a role for high MD in suppressing the inflammatory EF B cell response and probably preventing severe disease outcomes in patients with acute SARS‐CoV‐2 infection. Moreover, the extent of MD in B cells may serve as an indicator for disease severity.

### 2.9 Unvaccinated patients exhibited more EF‐like B‐cell activation and mitochondrial dysfunction than vaccinated APs

**FIGURE 7:**
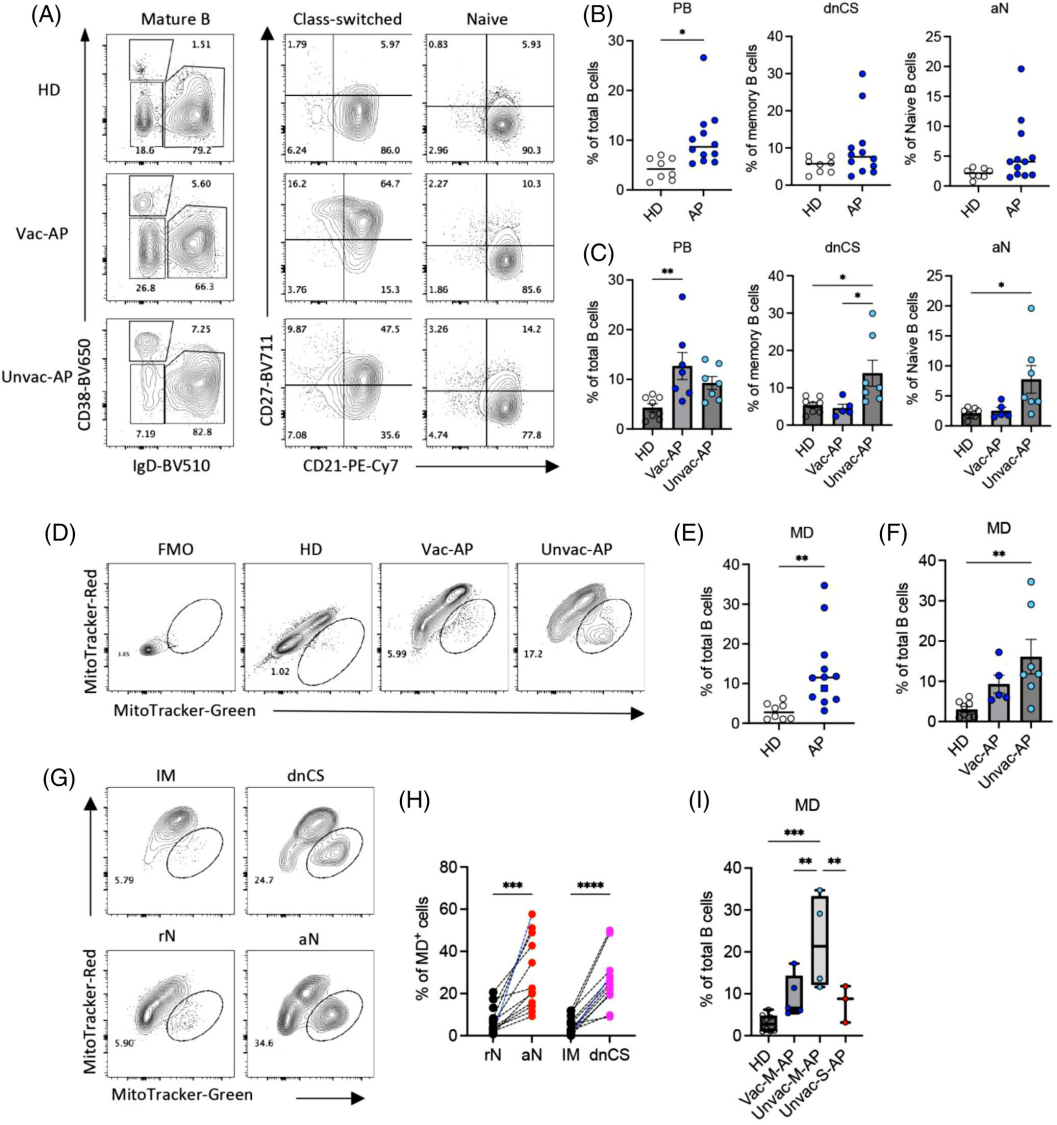
Patients without receiving COVID‐19 vaccine exhibited more excessive extrafollicular (EF)‐like B‐cell activation and mitochondrial dysfunction (MD) during acute infection. (A‐C) Per PBMCs from healthy donors (HDs) (*n* = 8), vaccinated acute patients (APs) (Vac‐AP, *n* = 7) and unvaccinated APs (Unvac‐AP, *n* = 5) were analysed by flow cytometry. Representative patient samples were selected for display (A). (A) Primary population (plasmablasts [PB], naïve and class‐switched B cells) and secondary population (activated classical memory [AM], resting memory [RM], double negative class‐switched [dnCS], intermediated memory [IM], activated naïve [aN] and resting naïve [rN]) gating of representative patient samples. (B and C) Comparison of PB, dnCS and aN frequencies among HDs, Vac‐AP and Unvac‐AP. (D–F) Comparison of the frequencies of MD^+^ B cells between HDs, Unvac‐AP and Vac‐AP. Part (D) displays MD^+^ cell gating of representative patient samples. (G and H) Comparison of MD in indicated subpopulations in APs. Part (G) displays MD^+^ cell gating of representative patient samples. (I) Comparison of the frequencies of MD^+^ B cells between HDs, Vac‐M‐AP, Unvac‐M‐AP and unvaccinated severe acute patient (Unvac‐S‐AP). All samples were from the third group of APs. Data represent two measurements. Statistical significance was determined using ordinary one‐way ANOVA and the two‐stage linear step‐up procedure of Benjamini, Krieger and Yekutieli, with a single pooled variance for (B, D and F), and paired *t*‐test for (G). *p ≤ .05, **p ≤ .01, ***p ≤ .001 and ****p ≤ .0001

COVID‐19 vaccines are effective in protection against severe disease. In January 2022, we recruited the third group of 12 APs and 8 HDs to investigate the B‐cell response in patients that received priors COVID‐19 vaccine. All APs were recruited within 14 days PSO. Among the 12 patients, 5 patients received 2 doses COVID‐19 vaccines (Vac‐AP), and 7 APs were unvaccinated (Unvac‐AP). All Vac‐APs and four Unvac‐APs were M‐APs (Vac‐M‐APs and Unvac‐M‐APs), and three Unvac‐APs were S‐APs (Unvac‐S‐APs). Using flow cytometry, we investigated EF response and MD in B cells. We found that APs recruited in 2022 also exhibited an expansion of PBs and MD^+^ B cells in comparison with HDs (Figure 7A,B,D,E). Moreover, MD was predominantly induced in EF‐like, dnCS and aN B cells (Figure 7G,H). However, the increase of dnCS and aN B cells did not reach statistical significance in APs in 2022 (Figure 7A,B). Further analysis revealed that a significant expansion of dnCS, aN and MD^+^ B cells was only observed in Unvac‐APs (*n* = 7) but not Vac‐APs (*n* = 5) (Figure 7A,C,D,F). Notably, among the Unvac‐APs, the M‐APs consistently showed higher MD in B cells than S‐APs (Figure 7I). All APs recruited in 2020 were not vaccinated. Therefore, these data were in‐line with the previous observation that unvaccinated patients with COVID‐19 displayed enhanced EF B‐cell activation, and M‐APs likely engaged MD to counteract excessive EF B‐cell activation. Interestingly, increased EF B‐cell expansion and MD was not observed in Vac‐M‐APs, suggesting that although SARS‐CoV‐2 preferentially induced EF response in unvaccinated individuals, COVID‐19 vaccination prior to infection was associated with predominate follicular response and decreased EF activation to SARS‐CoV‐2 and mild diseases.

## 3 DISCUSSION

In this study, we provided experimental evidence that the rapid change of mitochondrial function in B cells regulated pathogenic EF B‐cell responses associated with COVID‐19 severity, a previously unrecognized defence mechanism in response to acute SARS‐CoV‐2 infection. We found that SARS‐CoV‐2 infection induced rapid and predominant EF B‐cell responses during primary infection in unvaccinated patients, which associated with higher serum concentrations of antibody and inflammatory cytokine and disease severity. MD, however, was promptly induced in B cells within the first week PSO in M‐APs, which suppressed excessive EF B‐cell proliferation through the increased production of intracellular calcium, resulting in reduced antibody production but increased neutralizing potency index. COVID‐19 vaccination prior to infection was associated with lower EF B‐cell activation and mild disease. Our results, therefore, indicated that the host immune system engages in MD to counteract SARS‐CoV‐2‐induced pathogenic B‐cell responses, which have significant implications for COVID‐19 pathogenesis and therapeutic interventions.

Excessive EF B‐cell responses correlate with COVID‐19 severity during the acute SARS‐CoV‐2 infection. In response to viral infection, activated B cells may engage canonical and non‐canonical B‐cell responses. Canonical B‐cell responses often start with an initial wave of PBs arising from EF sites, followed by the GC reaction. In GCs, most activated B cells expressing low affinity or self‐recognizing BCRs are eliminated, whereas B cells with somatically mutated high affinity BCRs differentiate into memory B cells and antibody secreting cells. Non‐canonical B‐cell responses, however, feature predominant EF responses without the GC reaction. This situation has been documented among patients with *Ehrlichia muris*, *B. burgdorferi* and SLE.^24^, ^28^, ^40^ In this study, by analysing a total of 137 APs in Hong Kong, we observed that an early and robust expansion of EF B cell populations correlated with COVID‐19 severity in patients. In contrast, frequencies of follicular B‐cell populations did not differentiate significantly between mild and severe illnesses. These findings suggested that acute SARS‐CoV‐2 infection primarily induces predominant EF B‐cell responses. In‐line with our results, some critically ill patients displayed robust EF B‐cell responses.^23^ Moreover, a recent report indicated that increased TNF‐α concentration in spleens was a possible cause of the loss of GC formation in COVID‐19 patients.^9^ We also showed an enhanced downregulation of CXCR5 on rN B cells in S‐APs, which might contribute to a stronger expansion of EF populations due to reduced follicular homing. Inhibitions of the GC response by TNF‐α and depletion of CXCR5 was reported in mice.^55^, ^56^ Studies revealing the mechanisms underlying enhanced EF B‐cell responses in S‐APs are needed to help elucidate the COVID‐19 pathogenesis. Interestingly, although the third group of APs in 2022 was predominantly infected by SARS‐CoV‐2 Omicron variants, enhanced EF response was consistently observed in S‐APs, suggesting that the expansion of EF B cells correlated with disease severity rather than strain variation.

M‐APs engage in MD to counteract excessive EF B‐cell activation. Mitochondrion is an essential regulator of cellular energy and metabolism and plays a crucial role in regulating B‐cell activation, proliferation, differentiation and antibody production.^56^, ^57^, ^58^ The most prominent role of mitochondria is the synthesis of ATP. This process involves electron transport along electron transport chain complexes when protons are pumped across the inner mitochondrial membrane to create a proton gradient. Together with the proton gradient, mitochondrial membrane potential generated by proton pumps forms the transmembrane potential of hydrogen ions to drive the synthesis of ATP by oxidative phosphorylation. The loss of mitochondrial membrane potential results in MD and B‐cell apoptosis.^59^ To date, although a few publications have indicated the possible involvement of MD in regulating the cellular metabolic pathways of monocytes and T cells during SARS‐CoV‐2 infection,^60^, ^61^, ^62^, ^63^, ^64^ little is known about how MD affects B‐cell responses in patients with COVID‐19. Here, we found that the induction of MD in B cells occurred within the first week PSO primarily among M‐APs. During this process, MD mediated the suppression of EF B‐cell proliferation and related antibody production more significantly in M‐APs than in S‐APs as demonstrated by multiple experiments. Before this study, the cause of MD induction remains poorly investigated.

Several factors may account for MD induction in COVID‐19 patients. First, MD increases with ageing.^65^ This factor, however, was unlikely to be a direct cause of increased MD in COVID‐19 patients because M‐APs were often younger than S‐APs. We found that patients aged less than 60‐year old exhibited a significantly higher frequency of MD^+^ B cells than those older than 60 years. Second, many viruses hijack mitochondria for their survival, propagation or evasion of host immunity. For example, HIV‐1 RNA were detected in mitochondria and associated with a decrease in mitochondrial function.^66^, ^67^ Similarly, ORF8b of SARS‐CoV‐2 might cause ER stress, MD and caspase‐independent cell death.^68^ Third, SARS‐CoV‐2 spike glycoprotein alone induced MD in endothelial cells in blood vessels by downregulating ACE2 expression.^69^ In this study, we focused on the role of intracellular calcium in the regulation of MD.^41^, ^49^, ^50^, ^70^ We detected a significant increase of intracellular calcium, which strongly correlated with the magnitude of MD in B cells. Experimentally, we showed that neutralizing intracellular calcium by BAPTA reduced MD in B cells. These results indicated that MD was likely induced by a burst of calcium in B cell during acute SARS‐CoV‐2 infection. An excessive increase in intracellular calcium level can be induced by chronic BCR stimulations.^41^ This is supported by our finding that magnitude of MD^+^ B cells positively correlated with the SARS‐CoV‐2 nasal swab viral load in APs (Figure 5F). However, high MD and high intracellular calcium were observed not only in activated B cells, such as aN and dnCS, but also in rN and memory B cells, suggesting that this response is unlikely to be triggered by antigen‐specific immune events, such as BCR binding alone. Future studies are still needed to determine how SARS‐CoV‐2 infection causes excessive intracellular calcium production in such a broad range of B‐cell subsets.

Induction of MD in EF B cells increased anti‐S1 IgG neutralizing potency during acute SARS‐CoV‐2 infection. Highly potent NAbs are essential for protection against SARS‐CoV‐2 infection in animal models,^72^ which predict survival in COVID‐19 patients.^11^ In contrast, high level of antibodies with low neutralization potency correlated to increased pro‐inflammatory cytokines signature in severely ill patients.^11^ EF responses can rapidly produce NAbs with lower affinity. Production of highly potent NAbs requires GC reaction to ensure high affinity BCR selection.^18^ Acute SARS‐CoV‐2 infection, however, induced prolonged and enhanced EF response without much of GC reaction in severe patients.^9^, ^34^ The enhanced EF response and loss of GC often leads to higher frequency of antibodies with low affinity in the blood.^18^ This is in contrast to canonical response, which features GC reaction that produces potent NAbs after a short initial wave of EF response.^18^ The enhanced EF responses, therefore, are likely responsible for the lower NAbs potency observed in severely ill patients with COVID‐19.^11^ In this study, we found that M‐APs engaged in MD to suppress B‐cell responses. However, the MD was primarily found in EF population, which suppressed EF response predominantly. As a result, MD‐mediated suppression likely occurred more on EF‐derived antibodies, such as autoantibodies and antibody with lower affinities. This is supported by our findings that more significant differences between MD high and MD low groups were observed on the titre of anti‐SSA, anti‐dsDNA and anti‐RBD IgG antibodies than NAbs, Moreover, MD^+^ dnCS% positively correlated with anti‐S1 antibody neutralization potency in our patients.

Lack of MD in EF B cells correlated closely with lower autoantibody production and poorer disease outcomes. Recent studies reported higher serum titre of autoantibodies against cytokines, chemokines and complement components in that S‐APs, which perturbed host immune responses for viral control.^15^, ^16^, ^17^ In this study, we analysed the association of EF B‐cell populations with autoantibody production and disease severity, which have not been investigated previously with a large number of patient's samples. We tested immune tolerance by measuring the levels of two autoantibodies, anti‐SSA/Ro and anti‐dsDNA that commonly exist in individuals with B‐cell‐mediated autoimmune diseases. We found significantly elevated serum levels of both autoantibodies in COVID‐19 patients compared with HDs. Moreover, S‐APs presented significantly higher autoantibody serum titres than M‐APs. As GC response was largely suppressed in COVID‐19 patients, the increased autoantibody levels in S‐APs were more likely due to their enhanced EF B cell responses. Concordantly, the magnitude of EF B cell populations positively correlated with serum concentrations of both anti‐SSA/RO and anti‐dsDNA autoantibodies. In consistency with our findings, a recent study described increased amounts of serum 9G4‐idiotype autoantibodies in S‐APs.^23^

In addition, EF B cells may play antibody‐independent pathological roles mediated by the generation of inflammatory cytokines, such as IL‐6, IFN‐γ and TNF‐α.^24^, ^40^, ^73^ This is supported by our finding that increased EF B cells positively correlated with serum levels of IL‐6 and CRP. This is also consistent with the previous study that EF expansion correlated with elevated serum concentration of biomarkers of severe COVID‐19.^23^ We found that the increased magnitude of MD^+^ B cells correlated the increased serum concentration of IL‐6 and CRP. Critically, patients with a high frequency of MD^+^ B cells displayed significantly lower serum concentrations of autoantibodies, IL‐6 and CRP, suggesting the role of MD in suppressing pathogenic B‐cell response in COVID‐19 patients. This conclusion was further supported by the significant correlations between increased MD and specific clinical characteristics and disease severity (Table S4). In summary, our findings on the role of the mitochondria in regulating SARS‐CoV‐2‐induced B‐cell responses, and disease severity may have significant implications to COVID‐19 pathogenesis, therapeutic interventions and vaccine development.

### 3.1 Limitation of the study

Although our data demonstrated that elevated intracellular calcium caused MD in EF B cells in COVID‐19, the reasons for eliciting the increased calcium influx in the EF B‐cell subpopulations in COVID19 require further investigation. Previous reports demonstrated that although calcium signals are critical for the development of B‐cell response to the antigen, sustained elevation of intracellular calcium induces MD and promotes B‐cell apoptosis.^42^, ^74^ The elevated Ca^2+^ in the cytosol induced by BCR initially comes from the intracellular stores, endoplasmic reticulum. However, a sustained elevation of Ca^2+^ cytosolic level requires a prolonged influx of extracellular Ca^2+^, which is regulated by store‐operated calcium entry and calcium release‐activated calcium channels. BCR engagement by antigen binding activates PLCγ‐2 and IP_3_ receptors, through which calcium leaves endoplasmic reticulum, resulting in the depletion of endoplasmic reticulum Ca^2+^, and activation of store‐operated activation of calcium release‐activated calcium channels.^75^ Our study demonstrated that high MD was associated with higher viral load in APs. Whether chronic BCR stimulation by SARS‐CoV‐2 proteins caused elevated Ca^2+^ cytosolic level and MD is unknown.

Furthermore, recent studies suggested that severely ill patients had increased levels of IgG1 with afucosylated Fc glycans, which enhances its interaction with the Fcγ receptor FcγRIIIa and the production of inflammatory cytokines by monocytes.^12^ Other studies demonstrated that autoantibodies against cytokines, chemokines and compliment components perturbed the immune response and viral control in severely ill patients.^15^, ^76^, ^77^ Based on our findings, a lack of prompt MD in B cells correlated significantly with a stronger antibody response and severe COVID‐19. Whether MD is directly involved in suppressing production of antibodies with afucosylated Fc glycans or autoantibodies needs further investigation.

## 4 METHODS

### 4.1 Human subjects

In this study, we investigated a total of 137 APs infected with SARS‐CoV‐2 who were admitted to the Hong Kong Queen‐Mary Hospital and Princess Margaret Hospital during our study period between 17 July 2020 and 28 January 2022. The first group included 64 patients between 8 July and 6 August 2020 for primary experiments, whereas the second group of 61 patients and third group of 12 patients were recruited between 23 July 2020 and 7 December 2020 and 28 January 2022 for confirmation tests. Meanwhile, 13 samples of PBMCs collected from CPs (*n* = 9) and 43 samples from HDs were included as controls. The median age of CPs and HDs were 55 (interquartile range, 41–62) and 35 (interquartile range, 31–52), respectively. All patients were confirmed to be positive for SARS‐CoV‐2 by RT‐PCR.^26^ The first two groups of APs were recruited before the first report of identified case with SARS‐CoV‐2 Alpha (B.1.1.7) infection in Hong Kong.^78^ The third group of APs were recruited during a major wave predominantly of Omicron BA.2.2.^79^ APs were divided into mild and severe patient groups based on their requirement of oxygen supplementation. APs who required oxygen supplementation were considered S‐APs, the remaining APs were M‐APs.

### 4.2 Study design

All blood samples from APs were collected within 3 weeks after symptom onset. Plasma and PBMCs were obtained from whole blood. Seventy‐three PBMC samples were derived from the first group APs (*n* = 64) between 17 July and 6 August 2020, because nine APs had two samples at different week PSO. Freshly isolated PBMCs were stained and analysed using flow cytometry for B‐cell populations, activation status, mitochondrial function and homing potential and were compared with samples from CPs (*n* = 13) and HDs (*n* = 12) with similar age and sex distributions (Table S2). The B‐cell‐proliferative response (carboxyfluorescein‐6‐succinimidyl ester [CFSE] assay) was measured in parallel in 29 fresh PBMC samples from the first group of APs and 6 fresh PBMC samples from the second group of APs recruited at 23 July and 3 August 2020. Six PBMCs samples from six HDs were used as controls. From the 73 AP blood samples, all plasma samples were tested for cytokine concentration, and titre of NAbs and RBD‐specific IgG. After these tests, 20 plasma samples were used up. Remaining 53 plasma samples were tested for SSA/Ro‐, dsDNA, S1‐ and RBD‐specific IgG.

Subsequently, 14 PBMCs samples from 14 M‐APs were collected on 7 December 2020 to study the role of intracellular calcium in the induction of MD (Tables S1 and S2). To consolidate the EF B‐cell phenotype and mitochondria function, frozen PBMC samples from 17 HDs and 41 APs in July 2020 were recovered. Six APs and six HDs samples were stained for EF phenotype analysis by FACs (Panel 6). Five APs and five HDs samples were stained for IL‐6 cytokine expression analysis by FACs (Panel 7). B cells in 30 APs and 6 HDs were analysed for MD phenotype by flow cytometry (Panel 5) and then pooled and analysed by Seahorse XF Cell Mito Stress Test (Figure S4). Between 28 January and 3 March 2022, 12 APs were recruited to investigate B‐cell response in patients who received prior COVID‐19 vaccines. All samples were selected based on their availability. Data from each sample used to generate figures and tables are presented in Figure S8. The patient numbers and sample numbers for each assay are summarized in Table S2.

### 4.3 Peripheral blood mononuclear cell (PBMC) isolation

PBMCs were isolated from fresh blood samples collected from patients and HDs using Ficoll‐Paque‐density‐gradient centrifugation in our BSL‐3 laboratory. The majority of purified PBMCs were used for immune cell phenotyping and B‐cell isolation, whereas plasma samples were subjected to antibody and cytokines testing. The remaining cells were cryopreserved in freezing medium (90% FBS+10% DMSO) at a density of 5 × 10^6^ cells/ml at −150°C.

### 4.4 The 12‐colour flow cytometry analysis

Six panels of mAbs were used for the 12‐colour flow cytometry analysis (BioLegend, eBioscience and BD Biosciences) (Table S3). Cells were incubated for 10 min with Fc Block (Biolegend) in staining buffer (PBS containing 2% FBS) followed by staining with the indicated antibodies for 30 min at 4°C. For intracellular staining, cells were fixed and permeabilized with BD Cytofix/Cytoperm (BD Biosciences) prior to staining with mAbs against cytokines in Perm/Wash buffer (BD Biosciences) for 30 min at 4°C. Stained cells were washed with 1 ml of staining buffer and acquired using an FACSAria III flow cytometer (BD Biosciences) inside a BSL‐3 laboratory and analysed with FlowJo software (v10.6) (BD Biosciences).

For the analysis of mitochondrial function, after cells were washed with prewarmed glucose‐free RPMI 1640 medium (Gibco) supplemented with 10% FBS (staining buffer), fresh or frozen cells were resuspended in 100 μl of staining buffer containing 40‐nM MitoTracker Green FM (Invitrogen) and 40‐nM MitoTracker Red CMXRos (Invitrogen) and incubated in a CO_2_ incubator at 37°C for 30 min or 40‐nM MitoTracker Green FM and 125‐nM tetramethylrhodamine methyl ester (TMRM) at 37°C for 15 min. Stained cells were washed with 1 ml of prewarmed staining buffer and immediately used for FACS analysis. Cells were stained with 5‐μM Fluo‐4FF AM (Invitrogen) together with phenotyping antibodies at 4°C for 30 min to measure changes in cellular calcium concentrations. Data were acquired using an FACSAria III flow cytometer (BD Biosciences) inside a BSL‐3 laboratory and analysed with FlowJo software (v10.6) (BD Biosciences).

### 4.5 UMAP visualization of flow cytometric data

For UMAP projections, all samples stained with Panel 5 were gated for Zombie^−^CD19^+^ B cells and then downsampled to 150 cells using the DownSample plugin (v3.3) in FlowJo. M‐AP samples collected on days 0–6 (*n* = 26), days 7–13 (*n* = 22) and days 14–21 (*n* = 5), S‐AP samples collected on days 0–6 (*n* = 6), days 7–13 (*n* = 8) and days 14–21 (*n* = 6) after symptom onset, and HDs samples (*n* = 12) were concatenated to create seven composites, including M1, M2, M3, S1, S2, S3 and HD, respectively. Subsequently, 900 cells of each composite were tagged with disease states and were used to concatenate into a single 6300 CD19+ B‐cell composites. A UMAP algorithm for dimensionality reduction was applied using the UMAP plugin (v3.1) (Figure 3B). The composite sample was then re‐gated for primary populations, MD^+^ cells and patients with different disease states, M‐APs, S‐APs and HD, to aid in visual overlays in exploration of the UMAP projections (Figure S3a). Density plots with levels set to 10% of gated primary populations, MD^+^ cells, M‐APs and S‐APs populations were then projected onto the UMAP coordinates (Figures S3b and 3C,D,F). These UMAP projections were exported to be superimposed and processed for display. Overlapping densities were removed to reveal only densities occupied by S‐APs or M‐APs groups (Figure 3E,F). Subsequently, the densities occupied by only M‐APs (BCP1, BCP2, BCP4) or S‐APs (BCP3 and BCP5) were gated for primary and secondary populations (Figure 3H).

### 4.6 B‐cell isolation

Total B cells were isolated from human PBMCs using a pan B‐cell isolation kit (Miltenyi Biotec) followed by IgD‐positive selection using anti‐IgD microbeads (Miltenyi Biotec) according to the manufacturer's instructions.

### 4.7 Proliferation assay

CFSE (5 μM in PBS, Biolegend)‐labelled B cells were cultured in 96‐well U‐bottom plates with RPMI 1640 medium containing 10% FBS and 1% streptomycin/penicillin (all from Gibco) to measure B‐cell proliferation. B cells were then cultured in the presence of anti‐IgM/G/A (10 μg/ml, Invitrogen) and CpG (1 μM, Invitrogen) or anti‐IgM/G/A (10 μg/ml), IL‐2 (20 U/ml, R&D Systems), IL‐10 (100 ng/ml, R&D Systems) and CD40L (500 ng/ml, PeproTech) for 4 days. Proliferating B cells were determined by calculating the percentage of CFSE‐low cells.

### 4.8 B‐cell treatment

PBMCs were pretreated with or without BAPTA‐AM (25 μM, Invitrogen) in R10 (RPMI 1640 medium containing 10% FBS and 1% streptomycin/penicillin, Gibco) for 1 h at 37°C with 5% CO_2_ and then were washed three times with prewarmed medium and cultured in R10 for 24 h before flow cytometry analysis (Panel 5). For valinomycin treatment, 10‐μM to 1‐mM valinomycin (Invitrogen, catalogue V1644) was added to .5 × 10^6^ PBMCs or B cells. All aforementioned cells were maintained in a 37°C humidified incubator in an atmosphere of 5% CO_2_–95% air for 1 h or 24 h.

### 4.9 Extracellular flux assay

Seahorse XF96 Cell Mito stress test was performed as described in the manufacturer protocol. In brief, B cells of 30 APs and 3 HDs were isolated from the cryopreserved PBMC vials. B cells of 30 APs were pooled together to obtain sufficient signal. A total of .5 × 10^6^ purified B cells per well were resuspended in a 180‐μl Seahorse medium (unless otherwise specified) and plated in a 96‐well Cell‐Tak (Corning)‐coated Seahorse plate. Cells were maintained in 37°C in a non‐CO_2_ incubator for at least 1 h before the assay. Cells were treated sequentially with 1.5‐μM oligomycin (an inhibitor of ATP synthase), 10‐μM carbonyl cyanide‐*p*‐trifluoromethoxyphenylhydrazone (FCCP, an uncoupler of mitochondrial oxidative phosphorylation) and 5‐μM rotenone plus 5‐μM antimycin A (inhibitors of mitochondrial electron transport chain). Oxygen consumption rate (OCR) was measured using a Seahorse XF96 analyser.

### 4.10 Pseudotype viral neutralization assay

Plasma samples were inactivated at 56°C for 30 min prior to a pseudotype viral entry assay to determine the anti‐viral activity of patient plasma as previously described.^80^, ^81^, ^82^ Briefly, the SARS‐CoV‐2 pseudotype virus was generated through the cotransfection of 293T cells with two plasmids, pVax‐1‐S‐COVID19 and pNL4‐3Luc_Env_Vpr, carrying the optimized SARS‐CoV‐2 S gene and a human immunodeficiency virus type 1 backbone, respectively.^8^ At 48‐h post‐transfection, the viral supernatant was collected and frozen at −150°C. Serially diluted plasma samples were incubated with 200 TCID_50_ of pseudovirus at 37°C for 1 h. The plasma‐virus mixtures were then added to preseeded HEK293T‐hACE2 cells. After 48 h, infected cells were lysed, and luciferase activity was measured using Luciferase Assay System kits (Promega) in a Victor3‐1420 Multilabel Counter (PerkinElmer). The 50% inhibitory concentrations (IC_50_) of each plasma specimen were calculated to reflect anti‐SARS‐CoV‐2 potency.

### 4.11 Enzyme‐linked immunosorbent assay (ELISA)

An enzyme‐linked immunosorbent assay (ELISA) was performed to detect SARS‐CoV‐2 RBD‐specific IgG, as previously described.^3^ Briefly, 96‐well plates (Costar) were coated with recombinant SARS‐CoV‐2 RBD antigen (50 ng/well; Sino Biological) at 4°C overnight. After washes with PBST (.05% Tween‐20 in PBS), the plates were blocked with 4% skim milk in PBS for 1 h at 37°C and incubated with serially diluted patient plasma for 1 h at 37°C. After washes with PBST, goat anti‐human IgG conjugated with HRP (Santa Cruz Biotechnology) was added and incubated for 1 h, followed by washes and the addition of 50 ml of the HRP chromogenic substrate 3,3′,5,5′‐TMB (Sigma). Optical density (OD) values were measured at 450 nm using a VARIOSKANTM LUX multimode microplate reader (Thermo Fisher Scientific). A fivefold difference in the mean OD values detected from blank wells containing 4% skim milk in PBS alone was used as a cut‐off for the endpoint antibody titre calculation. For autoantibody detection, the SSA/Ro60274‐290 (QEMPLTALLRNLGKMT) peptide was synthesized (SBS Genetech Co., Ltd.). Serum levels of anti‐dsDNA and anti‐SSA/RO autoantibodies were determined using ELISAs as previously described.^82^, ^83^ All experiments were performed in duplicate.

### 4.12 Quantification of cytokine, anti‐RBD and S1 IgG concentration in serum

A panel of 13 cytokines, including IL‐1β, IFN‐α, IFN‐γ, TNF‐α, MCP1, IL‐6, IL‐8, IL‐10, IL‐12p70, IL‐17A, IL‐18, IL‐23 and IL‐33, and IgG against RBD and S1 protein were quantified in patient sera using multiplexing laser bead technology (BioLegend).

### 4.13 Quantification and statistical analysis

Statistical analyses were performed with GraphPad Prism 8 Software. Statistical significance between different groups was determined using ordinary one‐way ANOVA and the two‐stage linear step‐up procedure of Benjamini, Krieger and Yekutieli, with a single pooled variance, or a two‐tailed Student's *t*‐test. The Mann–Whitney *U* test was conducted for the data shown in Table S1. ROC analysis was performed with the Mann–Whitney *U* test and correlation analyses were performed with a linear regression model. A *p* value less than .05 was considered statistically significant. Data are presented as the mean values ± standard errors. Heat maps of the correlation coefficient matrices were generated using the R package pheatmap 1.0.12, and the analysis procedures were based on R 3.6.2.

## FUNDING INFORMATION

This work was partially supported by the Research Grants Council Collaborative Research Fund (C7156‐20G) of the Hong Kong Special Administrative Region. Our team was partially supported by the Hong Kong Health and Medical Research Fund (18171302 and 19180632), Hong Kong Research Grants Council (T11‐709/18‐N), Innovation and Technology Commission, Innovation and Technology Commission, General Research Fund (17122915), National Natural Science Foundation of China (81703119) and generous donations, including the Friends of Hope Education. We also thank the University Development Fund of the University of Hong Kong and Li Ka Shing Faculty of Medicine Matching Fund to HKU AIDS Institute.

## CONFLICT OF INTEREST

The authors declare no financial or commercial conflicts of interest.

## Supporting information

Figure S1 Inflammation markers and NAbs in the plasma of APs
Figure S2 Gating strategy and comparison of B‐cell populations in HDs, M‐APs, S‐APs and CPs
Figure S3 B‐cell phenotype in COVID‐19 patients by flow cytometry analysis
Figure S4 MD profile in B cells by flow cytometry and Seahorse extracellular flux analysis
Figure S5 UMAP visualization of flow cytometric data and mitochondrial dysfunction in broad B‐cell subsets in patients with COVID‐19
Figure S6 Proliferative response of gD^+^ and IgD^−^ B cells to follicular and EF stimulations
Figure S7 Increase of intracellular calcium results in increased MD in B cells of APs
Figure S8 Overview of study design
Table S1 Clinical characteristics of patients
Table S2 Number of patients and blood samples included in each assay
Table S3 Reagents for different assays
Table S4 Clinical characteristics of COVID‐19 patients with high and low magnitude of B‐cell mitochondrial dysfunction

## Data Availability

The data that support the findings of this study are available on request from the corresponding author. The data are not publicly available due to privacy or ethical restrictions.
