# Supplementary material for: Mitochondrial regulation of acute extrafollicular B‐cell responses to COVID‐19 severity
Source: Clin Transl Med. 2022 Sep 14;12(9):e1025. doi: 10.1002/ctm2.1025 (PMC9473490; doi:10.1002/ctm2.1025)
Supplement: Supplementary file 1 — Figure S1 Inflammation markers and NAbs in the plasma of APs Figure S2 Gating strategy and comparison of B‐cell populations in HDs, M‐APs, S‐APs and CPs Figure S3 B‐cell phenotype in COVID‐19 patients by flow cytometry analysis Figure S4 MD profile in B cells by flow cytometry and Seahorse extracellular flux analysis Figure S5 UMAP visualization of flow cytometric data and mitochondrial dysfunction in broad B‐cell subsets in patients with COVID‐19 Figure S6 Proliferative response of gD+ and IgD− B cells to follicular and EF stimulations Figure S7 Increase of intracellular calcium results in increased MD in B cells of APs Figure S8 Overview of study design Table S1 Clinical characteristics of patients Table S2 Number of patients and blood samples included in each assay Table S3 Reagents for different assays Table S4 Clinical characteristics of COVID‐19 patients with high and low magnitude of B‐cell mitochondrial dysfunction [file CTM2-12-0-s001.docx]

**Supplementary Fig. 1. Inflammation markers and Nabs in the plasma of APs.** a, Comparison of CRP, IL-6, IL-10, IFN-α, TNF-α and MCP-1 in plasma between M-APs and S-APs weekly intervals PSO. b,Comparison of the viral load in nasal swabs between M-APs and S-APs weekly intervals PSO. c, Comparison of RBD-specific IgG and Nab serum titers between M-APs and S-APs weekly intervals PSO. Plasma samples were from M-APs (days 0-6: n=26; days 7-13: n=22; days 14-21: n=5) and S-APs (days 0-6: n=6, days 7-13: n=8; days 14-21: n=6). Data represent 2 measurements of each patient sample. Statistical significance was determined using unpaired student *t*-test. *, *P* ≤ 0.05; **, *P* ≤ 0.01; ***, *P* ≤ 0.001.

**Supplementary Fig. 2. Gating strategy and comparison of B cell populations in HDs, M-APs, S-APs and CPs.** a. Gating strategy for B cell populations by flow cytometry (Panel 1-6). Live mature B cells (Zombie^-^ CD10^-^ CD19^+^ ) were analysed for primary population (CD38 ++ IgD^-,^ PB, IgD-CD38- class-switched memory B cells, IgD^+^CD27^−^ naïve B cells), and secondary population (CD21^+^CD27^+^ RM, CD21^-^CD27^+^ AM, CD21^+^CD27^-^ IM, CD21^-^CD27^-^ dnCS, CD21^-^ aN and CD21^+^ rN). b. Comparison of B cell populations between HDs, CPs, M-APs and S-APs weekly intervals PSO. Seventy-three samples of PBMCs were from HDs (n=12), Cps (n=13), M-APs (days 0-6: n=26; days 7-13: n=22; days 14-21: n=5) and S-APs (days 0-6: n=6, days 7-13: n=8; days 14-21: n=6). Data represent 5 measurements of each patient sample in a and b, and two measurement in c. Statistical significance was determined using ordinary one-way ANOVA and the two-stage linear step-up procedure of Benjamini, Krieger and Yekutieli, with a single pooled variance. *, *P* ≤ 0.05; **, *P* ≤ 0.01; ***, *P* ≤ 0.001.

**Supplementary Fig. 3: B cell phenotype in COVID-19 patients by flow cytometry analysis.** a, Homing receptor surface expression in follicular (rN and MI) verses EF (aN and dnCS) populations in M- and S-APs. b, CD11c and FcRL5 surface expression in IM verses dnCS cells in APs (n=6) and HDs (n=6). c, Chemokine and cytokine receptor surface expression in rN B cells of HDs (n=12), M-APs (day 7-13: n=22; day 14-21: n=5)and S-APs (day 7-13: n=8; day 14-21: n=6). Data represent one measurement of each patient sample. Statistical significance was determined using unpaired *t*-test for a, paired t-test for b, and ordinary one-way ANOVA and Two-stage linear step-up procedure of Benjamini, Krieger and Yekutieli, with a single pooled variance for e. *, *P* ≤ 0.05; **, *P* ≤ 0.01; ***, *P* ≤ 0.001; ****, *P* ≤ 0.0001.

**Supplementary Fig. 4.** **MD profile in B cells by flow cytometry and Seahorse extracellular flux analysis.** a-c, Freshly isolated PBMCs from HDs (n=3) were treated with 10 µM, 50uM, 100uM, 500uM and 1000uM valinomycin for 24 hours or left untreated as control. a, Representative samples showing the percentage of MD^+^ B cells by MitoTracker™ Green (Mito Green) and MitoTracker™ Red CMXRos (Mito Red) or Mito Green and TMRM staining and flow cytometry analysis. b, Data represents Mean ± SEM. c, The correlation between these two indicators in valinomycin treated HD samples. d-f, Purified total B cells from fresh PBMCs of 3 HDs were treated with or without 10 µM Valinomycin for 60 min. d, The percentage of MD^+^ B cells detected by Mito Green and MitoRed staining and flow cytometry analysis. e-f, After performing Seahorse XF Cell Mito Stress Test, the oxygen consumption rate (OCR) along experimental time, ATP production and maximal respiration was analyzed and compared between valinomycin-treated group and non-treated control group. Data represents Mean ± SEM. g-i, Frozen PBMCs of 30 APs and 3 HDs were recovered. B cells were isolated for Seahorse XF Cell Mito Stress Test. g, The percentage of live cells and MD^+^ B cells was detected using Zombie, Mito Green and Mito Red staining and flow cytometry analysis (n=30). h-i, OCR along experimental time, Basal respiration, maximal respiration, Spare Respiration Capacity and ATP production was analyzed and compared between APs (n=30), valinomycin-treated group (n=3) and non-treated control group (n=3). Data represents Mean ± SEM. Statistics were calculated using paired Student’s t-test for f, unpaired *t*-test for g, and ordinary one-way ANOVA and the two-stage linear step-up procedure of Benjamini, Krieger and Yekutieli, with a single pooled variance for i. *, *P* ≤ 0.05; **, *P* ≤ 0.01; ***, *P* ≤ 0.001; and ****, *P* ≤ 0.0001. Correlation analyses were performed with a linear regression model using GraphPad Prism 8.0 software.

**Supplementary Fig. 5. UMAP visualization of flow cytometric data and mitochondrial dysfunction in broad B cell subsets in patients with COVID-19.** a. gating strategy for the composite sample from all patients for primary population (PB, unswitched and class-switched B cells) and MD^+^ B cells, or patient groups with different disease states (M-APs and S-APs) using Group ID. b. The UMAP projections of M-APs and S-APs were superimposed for display. c. Gating on MitoGreen^high^ MitoRed^low^ B cells to identify MD^+^ cells in representative HD, M-APs and S-APs. d, Frequencies of MD^+^ B cell subsets in HDs, M-APs and S-APs at weekly intervals PSO. e, MD+ cells frequency in IM (black) verses dnCS (pink), and rN (black) verses aN (red) cells in APs (n=73). f, Comparison of Zombie^-^ live B cells in HDs, M-APs and S-APs at weekly intervals PSO. Representative HD, M-AP and S-AP samples were selected for display (left). Samples in d, e and f were from HDs (n=12) and APs (n=73), including M-AP samples on days 0-6 (n=26), days 7-13 (n=22), and days 14-21 (n=5), S-AP samples on days 0-6 (n=6), days 7-13 (n=8), and days 14-21 (n=6) PSO. Data represent a single measurement from each patient time point assessed. Statistical significance was determined using ordinary one-way ANOVA and the two-stage linear step-up procedure of Benjamini, Krieger and Yekutieli, with a single pooled variance for d and f, and paired *t*-test for e. *, *P* ≤ 0.05; **, *P* ≤ 0.01; ***, *P* ≤ 0.001; and ****, *P* ≤ 0.0001.

**Supplementary Fig. 6: Proliferative response of gD^+^ and IgD^-^ B cells to follicular and EF stimulations.** a, Purification of IgD^+^ and IgD^-^ B cells. Frequencies of IgD^+^ and IgD^-^ B cells before and after B cell isolation were detected by Flow cytometry analysis. b, Representative patient samples were selected for display. Proliferated B cells were identified by the percentage of CFSE^low^ cells. c. Correlations of B cells proliferative capacity with frequencies of MD^+^IgD^+^ and MD^+^ IgD^-^ B cells in APs (n=29), respectively. d, Comparison of B cells proliferative response to follicular and EF stimulations between HDs (n=6), M-APs (n=28) and S-APs (n=7) before and after 7 days PSO. Patients samples included M-AP samples on days 0-6 (n=17) and days 7-21 (n=11), and S-AP samples on days 0-6 (n=2) and days 7-21 (n=5) PSO. Data represent a single measurement from each patient time point assessed. Statistical significance was determined using ordinary one-way ANOVA and the two-stage linear step-up procedure of Benjamini, Krieger and Yekutieli, with a single pooled variance for d. *, *P* ≤ 0.05; **, *P* ≤ 0.01; ***, *P* ≤ 0.001; and ****, *P* ≤ 0.0001. Correlation analyses were performed with a linear regression model using GraphPad Prism 8.0 software.

**Supplementary Fig. 7: Increase of intracellular calcium results in increased MD in B cells of APs**. a, Gating strategy for Fluo-4FF staining in B cell subtypes. Representative patient samples were selected for display. b, Frequencies of Fluo-4FF^high^ B cell subtypes were compared between HDs (n=12), M-APs at day 0-6 (n=26), day 7-13 (n=22), day 14-21 (n=5), and S-APs at day 0-6 (n=6), day 7-13 (n=8), day 14-21 (n=6) PSO. c. Correlation of B cell proliferation rate with Fluo-4FF high B cells frequency in APs (n=29). d, Gating strategy for live and mature B cells in PBMCs after BAPTA treatment. Representative patient samples were selected for display. e, Comparison of percentage of Zombie^-^ live cells in PBMCs of M-APs (n=14) with and without BAPTA treatment (25uM, 1hr). Data represent a single measurement from each patient time point assessed. Statistical significance was determined using ordinary one-way ANOVA and the two-stage linear step-up procedure of Benjamini, Krieger and Yekutieli, with a single pooled variance for b, and paired *t*-test for e. *, *P* ≤ 0.05; **, *P* ≤ 0.01; ***, *P* ≤ 0.001; and ****, *P* ≤ 0.0001. Correlation analyses were performed with a linear regression model using GraphPad Prism 8.0 software.

**Supplementary Fig. 8: Overview of study design**. A total of 137 APs infected with SARS-CoV-2 were studied. The first group included 64 APs between July 8 and August 6, 2020 for primary experiments, whereas the second group of 61 APs were between July 23 and December 07, 2020 for confirmation tests. All blood samples were collected within three weeks PSO. PBMCs and plasma were obtained. a, From the 64 APs, 73 PBMCs were collected because 9 APs had two samples at different week PSO. Freshly isolated PBMCs were analyzed by flow cytometry for B cell subsets, activation, homing potential and MD (Panel 1-5). 13 PBMCs from 9 CPs and 12 PBMCs from 12 HDs in July and August 2020 were included as controls. b, Proliferation assays were performed in parallel using 29 fresh PBMC samples from the first group and 6 fresh PBMC samples from the 2^nd^ group of APs. 6 PBMCs of HDs were used as controls. c, From the 73 AP blood samples in the 1^st^ group, all plasma samples were tested for cytokine, Nabs and RBD-specific IgG levels. Two heatmaps were generated using AP samples with data a and b (n=29) and data a and c (n=73), respectively. d, 53 plasma samples in group 1 were tested for S1-, RBD-, SSA- and dsDNA-specific IgG. e-f, To consolidate the EF B cell phenotype and MD, 41 frozen PBMC samples from APs in July 2020 were recovered. 6 and 5 samples were used for EF B cell phenotype consolidation and IL-6 expression, respectively, by FACs analysis (e, Panel 6 and 7). 30 samples were measured for MD by flow cytometry analysis (Panel 5), and subsequently pooled for Seahorse XF Cell Mito Stress Test (f). g, Freshly isolated PBMCs from 14 M-APs of 2^nd^ group on December 7^th^, 2020, were tested for BAPTA treatment. h, Freshly isolated PBMCs from 12 APs in 2022 were analyzed by flow cytometry for B cell subsets and MD analysis (Pane 5). All samples were selected based on their availabilities.

**Supplementary Table 1**. Clinical characteristics of patients

| **Characteristics** | **Severe (n=15)** | **Mild (n=49)** | **P value** |
| --- | --- | --- | --- |
| **Demographic** |  |  |  |
| Age, median years (interquartile range) | 77 (64-86) | 56 (41-64) | <0.001 |
| Female | 9 (60) | 22 (44.9) | 0.382 |
|  |  |  |  |
| **Chronic comorbidities** |  |  |  |
| Hypertension | 9 (60) | 10 (20.4) | 0.008 |
| Chronic heart disease | 3 (20) | 6 (12.2) | 0.427 |
| Chronic lung disease | 2 (13.3) | 1 (2.0) | 0.134 |
| Chronic liver disease | 0 (0) | 1 (2.0) | 1.000 |
| Chronic kidney disease | 1 (6.7) | 0 (0) | 0.234 |
| Diabetes mellitus | 2 (13.3) | 6 (12.2) | 1.000 |
| Malignancy | 1 (6.7) | 4 (8.2) | 1.000 |
| Chronic comorbidities | 13 (86.7) | 24 (49) | 0.015 |
|  |  |  |  |
| **Blood tests on admission**, (median, interquartile range) |  |  |  |
| Haemoglobin (g/dL) | 13.0 (11.4-14) | 13.6 (12.4-14.6) | 0.128 |
| Total white blood cell count (×10^9^/L) | 5.8 (4.7-8.9) | 4.9 (4.3-5.8) | 0.103 |
| Neutrophil count (×10^9^/L) | 4.7 (2.8-6.4) | 3.1 (2.5-3.7) | 0.053 |
| Lymphocyte count (×10^9^/L) | 0.8 (0.7-1.1) | 1.3 (0.9-1.7) | 0.018 |
| Platelet count (×10^9^/L) | 186 (136-239) | 229 (175-282) | 0.073 |
| Urea (mmol/L) | 5.4 (4.4-7.1) | 3.6 (3.0-4.8) | <0.001 |
| Creatinine (μmol/L) | 86 (72-105) | 69 (61-81) | 0.006 |
| Alanine aminotransferase (U/L) | 20 (14-37) | 23 (15-38) | 0.861 |
|  |  |  |  |
| **Severity** |  |  |  |
| Oxygen supplementation | 15 (100) | 0 (0) | <0.001 |
| ICU admission | 3 (20) | 0 (0) | 0.011 |
| Death | 5 (33.3) | 0 (0) | <0.001 |

Abbreviations: ICU, Intensive care unit

**Supplementary table 2. Number of patients and blood samples included in each assay**

| **Date of recruitments (patients No.)** | **Disease status and assays tested** | **AP** | | | | | | | | | | | | **CP** | | **HD** |
| --- | --- | --- | --- | --- | --- | --- | --- | --- | --- | --- | --- | --- | --- | --- | --- | --- |
|  |  | PBMC and plasma samples (n=146) | | | | | | | Patients (n=137) | | | | | PBMC (n=13) | Patient (n=9) | PBMC  (n=43) |
|  | Days after symptom onset | 0-6 | | 7-13 | | 14-21 | | Total | Unvaccinated | | Vaccinated | | Total |  |  |  |
|  | Severity of illness | Mild | Severe | Mild | Severe | Mild | Severe |  | Mild | Severe | Mild | Severe |  |  |  |  |
| Jul-Aug, 2020 (n=64) | Flow Cytometry (Panel 1) | 26 | 6 | 2 | 8 | 5 | 6 | 73 | 49 | 15 | 0 | 0 | 64 | 13 | 9 | 12 |
|  | Flow Cytometry (Panel 2-5), cytokines concentration, Nabs and RBD Ig titers in plasma | 26 | 6 | 22 | 8 | 5 | 6 | 73 | 49 | 15 | 0 | 0 | 64 | 0 | 0 |  |
|  | Anti-RBD/S1, Anti-dsDNA/SSA/Ro IgG1 | 22 | 4 | 12 | 8 | 3 | 4 | 53 | 37 | 16 | 0 | 0 | 53 | 0 | 0 |  |
|  | CFSE assay | 14 | 2 | 9 | 3 | 1 | 0 | 29 | 24 | 5 | 0 | 0 | 29 | 0 | 0 | 6 |
| Jul-Dec, 2020 (n=61) | CFSE assay | 0 | 1 | 4 | 0 | 1 | 0 | 6 | 4 | 2 | 0 | 0 | 6 | 0 | 0 | 0 |
|  | Flow Cytometry (Panel 6) | 6 | 0 | 0 | 0 | 0 | 0 | 6 | 6 | 0 | 0 | 0 | 6 | 0 | 0 | 6 |
|  | Flow Cytometry (Panel 7) | 5 | 0 | 0 | 0 | 0 | 0 | 5 | 5 | 0 | 0 | 0 | 5 | 0 | 0 | 5 |
|  | Flow Cytometry (panel 5) and Seahorse assay | N/A | N/A | N/A | N/A | N/A | N/A | 30 | N/A | N/A | 0 | 0 | 30 | 0 | 0 | 6 |
|  | MD analysis after BAPTA treatment | 6 | 1 | 6 | 1 | 0 | 0 | 14 | 14 | 0 | 0 | 0 | 14 | 0 | 0 | 0 |
| January, 2022 (n=12) | Flow Cytometry (Panel 5) | 8 | 3 | 0 | 0 | 0 | 0 | 12 | 4 | 3 | 5 | 0 | 12 | 0 | 0 | 8 |

N/A: data not available.

**Supplementary table 3: Reagents for different assays**

| **Flow Cytometry PBMC cell staining Assays** | **Source** | **Cat number** |
| --- | --- | --- |
| **Panel 1** |  |  |
| Human Fc Receptor Blocking Solution | Biolegend | Cat# 422302 |
| BV785 anti-human CD19 | Biolegend | Cat# 302240 |
| APC/Cy7 anti-human CD10 | Biolegend | Cat# 312212 |
| BV650 anti-human CD38 | Biolegend | Cat# 356620 |
| PE/Cy7 anti-human CD21 | Biolegend | Cat# 354912 |
| BV711 anti-human CD27 | Biolegend | Cat# 356430 |
| BV510 anti-human IgD | Biolegend | Cat# 348220 |
| Zombie Red Fixable Viability Kit | Biolegend | Cat# 423110 |
| BV421 anti-human CD183 (CXCR3) | Biolegend | Cat# 353716 |
| PerCP/Cy5.5 anti-human CD185 (CXCR5) | Biolegend | Cat# 356910 |
| PE anti-human CD279 (PD-1) | Biolegend | Cat# 329906 |
| **Panel 2** |  |  |
| Human Fc Receptor Blocking Solution | Biolegend | Cat# 422302 |
| BV785 anti-human CD19 | Biolegend | Cat# 302240 |
| APC/Cy7 anti-human CD10 | Biolegend | Cat# 312212 |
| BV650 anti-human CD38 | Biolegend | Cat# 356620 |
| PE/Cy7 anti-human CD21 | Biolegend | Cat# 354912 |
| BV711 anti-human CD27 | Biolegend | Cat# 356430 |
| BV510 anti-human IgD | Biolegend | Cat# 348220 |
| Zombie Red Fixable Viability Kit | Biolegend | Cat# 423110 |
| BV421 anti-human CD360 (IL-21R) | Biolegend | Cat# 359510 |

**Supplementary table 3: Reagents for different assays**

| **Flow Cytometry PBMC cell staining Assays** | **Source** | **Cat number** |
| --- | --- | --- |
| PE anti-human CD197 (CCR7) | Biolegend | Cat# 353204 |
| AF488 anti-mouse/human Ki-67 | Biolegend | Cat# 151204 |
| **Panel 3** |  |  |
| Human Fc Receptor Blocking Solution | Biolegend | Cat# 422302 |
| BV785 anti-human CD19 | Biolegend | Cat# 302240 |
| APC/Cy7 anti-human CD10 | Biolegend | Cat# 312212 |
| BV650 anti-human CD38 | Biolegend | Cat# 356620 |
| PE/Cy7 anti-human CD21 | Biolegend | Cat# 354912 |
| BV711 anti-human CD27 | Biolegend | Cat# 356430 |
| BV510 anti-human IgD | Biolegend | Cat# 348220 |
| Zombie Red Fixable Viability Kit | Biolegend | Cat# 423110 |
| PE anti-human CD184 (CXCR4) | Biolegend | Cat# 306506 |
| Fluo-4FF | Invitrogen | Cat# F23981 |
| **Panel 4** |  |  |
| Human Fc Receptor Blocking Solution | Biolegend | Cat# 422302 |
| BV785 anti-human CD19 | Biolegend | Cat# 302240 |
| APC/Cy7 anti-human CD10 | Biolegend | Cat# 312212 |
| BV650 anti-human CD38 | Biolegend | Cat# 356620 |
| PE/Cy7 anti-human CD21 | Biolegend | Cat# 354912 |
| BV711 anti-human CD27 | Biolegend | Cat# 356430 |
| BV510 anti-human IgD | Biolegend | Cat# 348220 |
| Zombie Red Fixable Viability Kit | Biolegend | Cat# 423110 |
| APC anti-human CD69 | Biolegend | Cat# 310910 |

**Supplementary table 3: Reagents for different assays**

| **Flow Cytometry PBMC cell staining Assays** | **Source** | **Cat number** |
| --- | --- | --- |
| PE anti-human CD80 | Biolegend | Cat# 305208 |
| PerCP/Cy5.5 anti-human CD86 | Biolegend | Cat# 374216 |
| **Panel 5** |  |  |
| Human Fc Receptor Blocking Solution | Biolegend | Cat# 422302 |
| BV785 anti-human CD19 | Biolegend | Cat# 302240 |
| APC/Cy7 anti-human CD10 | Biolegend | Cat# 312212 |
| BV650 anti-human CD38 | Biolegend | Cat# 356620 |
| PE/Cy7 anti-human CD21 | Biolegend | Cat# 354912 |
| BV711 anti-human CD27 | Biolegend | Cat# 356430 |
| BV510 anti-human IgD | Biolegend | Cat# 348220 |
| Zombie Violet Fixable Viability Kit | Biolegend | Cat# 423114 |
| MitoTracker Red CMXRos | Invitrogen | Cat# M7512 |
| MitoTracker Green FM | Invitrogen | Cat# M7514 |
| **Panel 6** |  |  |
| Human Fc Receptor Blocking Solution | Biolegend | Cat# 422302 |
| BV785 anti-human CD19 | Biolegend | Cat# 302240 |
| BV650 anti-human CD38 | Biolegend | Cat# 356620 |
| PE/Cy7 anti-human CD21 | Biolegend | Cat# 354912 |
| BV711 anti-human CD27 | Biolegend | Cat# 356430 |
| BV510 anti-human IgD | Biolegend | Cat# 348220 |
| Zombie Violet Fixable Viability Kit | Biolegend | Cat# 423114 |
| FITC anti-human CD71 | Biolegend | Cat# 334104 |
| PE anti-human CD307e (FcRL5) | Biolegend | Cat# 340304 |

**Supplementary table 3: Reagents for different assays**

| **Flow Cytometry PBMC cell staining Assays** | **Source** | **Cat number** |
| --- | --- | --- |
| PerCP/Cy5.5 anti-human CD185 (CXCR5) | Biolegend | Cat# 356910 |
| APC/Cy7 anti-human IgM | Biolegend | Cat# 314520 |
| APC anti-human CD11c | Biolegend | Cat#337208 |
| **Panel 7** |  |  |
| Human Fc Receptor Blocking Solution | Biolegend | Cat# 422302 |
| BV785 anti-human CD19 | Biolegend | Cat# 302240 |
| APC/Cy7 anti-human CD10 | Biolegend | Cat# 312212 |
| BV650 anti-human CD38 | Biolegend | Cat# 356620 |
| PE/Cy7 anti-human CD21 | Biolegend | Cat# 354912 |
| BV711 anti-human CD27 | Biolegend | Cat# 356430 |
| BV510 anti-human IgD | Biolegend | Cat# 348220 |
| Zombie Violet Fixable Viability Kit | Biolegend | Cat# 423114 |
| Percp cy5.5 IL-6 |  |  |
| **Proliferation Detecting Assays** |  |  |
| Goat anti-Human IgG, IgM, IgA (H+L) | Invitrogen | Cat# A24496 |
| Class B CpG | Invivogen | Cat# tlrl-2006 |
| Recombinant Human sCD40 Ligand​ | Peprotech | Cat# 310-02 |
| Recombinant Human IL-10 Protein | R&D systems | Cat# 217-IL |
| Recombinant Human IL-2 Protein | R&D systems | Cat# 202-IL |
| CFSE Cell Division Tracker Kit | Biolegend | Cat# 423801 |
| Human Fc Receptor Blocking Solution | Biolegend | Cat# 422302 |
| Zombie Violet Fixable Viability Kit | Biolegend | Cat# 423114 |
| APC anti-human CD69 | Biolegend | Cat# 310910 |

**Supplementary table 3: Reagents for different assays**

| **Neutralization Assays** | **Source** | **Cat number** |
| --- | --- | --- |
| BAPTA, AM, cell permeant chelator | Invitrogen | Cat# B6769 |

**Supplementary table 4. Clinical characteristics of COVID-19 patients with high & low magnitude of B cell mitochondrial dysfunction**

|  | Low frequency  group  (<23%, n=37) | High frequency group  (>23%, n=27) | P value |
| --- | --- | --- | --- |
| **Demographic** |  |  |  |
| Age, median years (interquartile range) | 64 (56-71) | 49 (38-60) | < 0.001 |
| Female | 20 (54.1) | 11 (40.7) | 0.322 |
|  |  |  |  |
| **Chronic comorbidities** |  |  |  |
| Hypertension | 12 (43.2) | 3 (11.1) | 0.006 |
| Chronic heart disease | 6 (16.2) | 3 (11.1) | 0.722 |
| Chronic lung disease | 3 (8.1) | 0 (0) | 0.257 |
| Chronic liver disease | 1 (2.7) | 0 (0) | >0.999 |
| Chronic kidney disease | 1 (2.7) | 0 (0) | >0.999 |
| Diabetes mellitus | 6 (16.2) | 2 (7.4) | 0.450 |
| Malignancy | 3 (8.1) | 2 (7.4) | >0.999 |
| Chronic comorbidities | 26 (70.3) | 11 (40.7) | 0.023 |
|  |  |  |  |
| **Blood tests on admission**, (median, interquartile range) |  |  |  |
| Haemoglobin (g/dL) | 13.0 (11.3-13.9) | 13.8 (13.5-14.7) | 0.007 |
| Total white blood cell count (×10^9^/L) | 5.48 (4.36-6.35) | 4.73 (4.31-5.53) | 0.116 |
| Neutrophil count (×10^9^/L) | 3.39 (2.77-4.37) | 2.96 (2.30-3.37) | 0.094 |
| Lymphocyte count (×10^9^/L) | 1.11 (0.75-1.76) | 1.25 (0.85-1.55) | 0.895 |
| Platelet count (×10^9^/L) | 234 (162-284) | 190 (174-249) | 0.181 |
| Urea (mmol/L) | 4.8 (3.6-6.5) | 3.6 (3.0-3.8) | 0.002 |
| Creatinine (μmol/L) | 72 (56-95) | 70 (64-75) | 0.366 |
| Alanine aminotransferase (U/L) | 23 (15-33) | 19 (17-41) | 0.615 |
|  |  |  |  |
| **Severity** |  |  |  |
| Oxygen supplementation | 14 (37.8) | 1 (3.7) | 0.002 |
| ICU admission | 3 (8.1) | 0 (0) | 0.257 |
| Death | 5 (13.5) | 0 (0) | 0.068 |

Abbreviations: ICU, Intensive care unit
